# Revisiting the Hunter-Sanders Model for π–π Interactions

**DOI:** 10.1021/jacs.5c03169

**Published:** 2025-05-29

**Authors:** Steven E. Wheeler

**Affiliations:** Department of Chemistry, 1355 University of Georgia, Athens, Georgia 30602, United States

## Abstract

The “Hunter-Sanders model” (*J. Am. Chem. Soc.* 1990, **112**, 5525) is foundational to many chemists’ understanding of interactions between aromatic systems. Carter-Fenk and Herbert (*Chem. Sci*., 2020, **11**, 6758) recently upended that understanding by showing that the driving force for aromatic systems to adopt parallel displaced geometries arises from steric, not Coulombic, repulsion of the π-electron clouds. Carter-Fenk and Herbert also claimed to show that the original Hunter-Sanders *potential* fails to predict the geometries of a range of parallel and T-shaped dimers. Closer inspection reveals that the data supporting this latter claim are flawed. Correctly implemented, the Hunter-Sanders potential provides qualitatively correct predictions for these systems and performs particularly well for the T-shaped benzene dimer. Moreover, it predicts the preferred displacement direction for some stacked heterocyclic dimers and accurately captures the impact of a diverse group of substituents on the benzene sandwich dimer. This is inclusive of the fact that all substituents enhance stacking interactions in this geometry. Ironically, for substituted benzene dimers, the Hunter-Sanders potential provides data in accord with our Local, Direct Interaction Model but in contrast with the so-called “Hunter-Sanders model.” At the same time, the Hunter-Sanders potential struggles to capture heteroatom and substituent effects in parallel displaced geometries in which the heteroatom/substituent is located over the other ring, leading to qualitatively incorrect predictions of the preferred displacement direction of substituted benzene dimers. Overall, many aspects of the Hunter-Sanders potential are flawed; however, others appear qualitatively correct.

## Graphical abstract

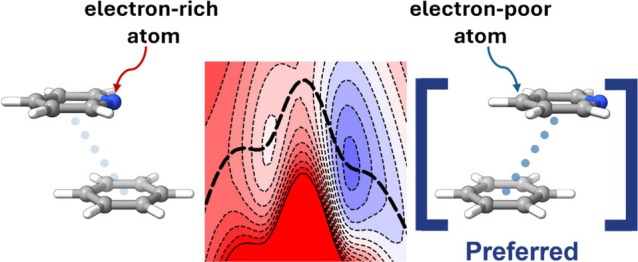

## 1 Introduction

Noncovalent interactions between aromatic rings are central to many areas of chemistry and biology.^1–3^ Understanding how to tune the strength and preferred geometry of these interactions undergirds the design of everything from organic electronic and stimuli-responsive materials^4–7^ to asymmetric catalysts^8–10^ and pharmaceuticals.^11–21^

Interactions between planar aromatic systems can be generally classified into sandwich (or eclipsed) stacking, parallel-displaced stacking, and T-shaped interactions (see Figure 1).^22^ For most dimers, parallel-displaced stacking is preferred over the eclipsed configuration, which is typically a saddle point on the respective potential energy surface. For benzene, the T-shaped configuration is slightly more favorable than the parallel-displaced dimer,^23^ while a slightly tilted (Cs-symmetric) T-shaped dimer is lower in energy still.^24,25^ For larger systems, the parallel-displaced configuration becomes increasingly energetically preferred.^26,27^

**Figure 1:**
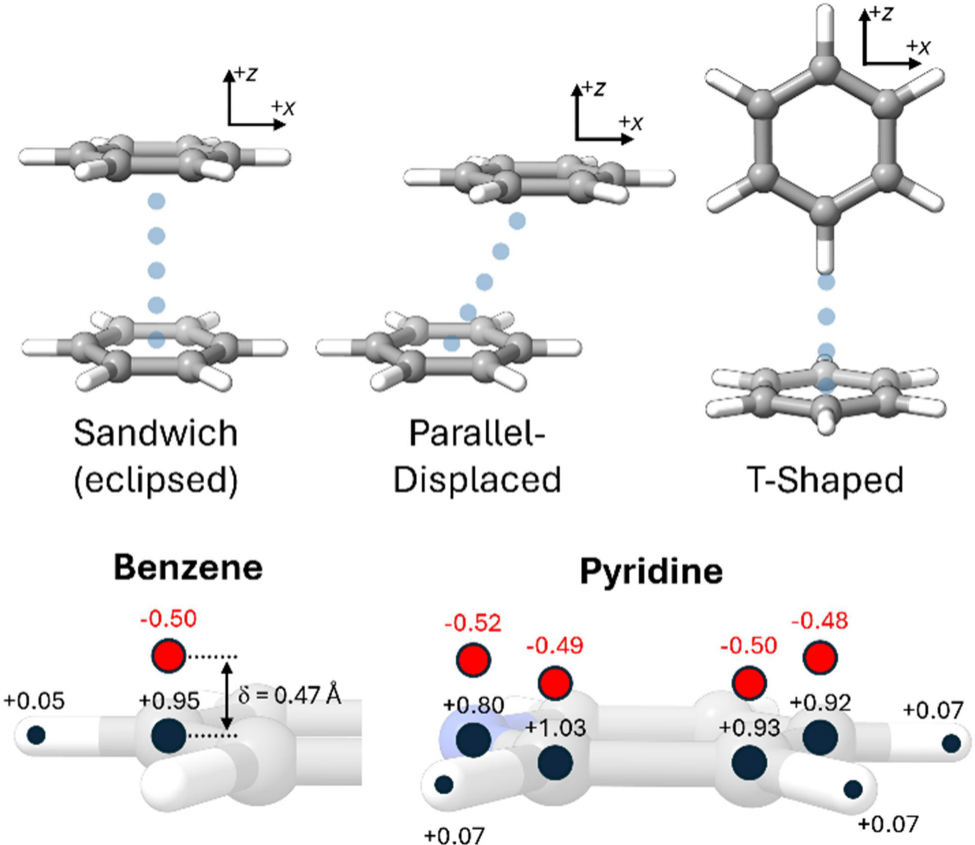
(top) Prototypical aromatic interactions as exemplified by the benzene dimer; directions of horizontal (*x*) and vertical (*z*) displacements indicated; (bottom) σ- and π-charges used for benzene^29^ and pyridine in *E*_elec_^HS^ [note that only one of each equivalent pair of π-charges (red) is shown].

In 1990, Hunter and Sanders published^28^ a groundbreaking study that has had lasting impact on discussions of the interactions of aromatic systems. First, they introduced “A Model for π–π Interactions” in the form of the pairwise interaction potential between molecules *A* and *B* in eq 1:

$$E_{int}^{HS}=E_{elec}^{HS}+E_{vdW}^{HS}=\underset{i\in A}{\sum }\underset{j\in B}{\sum }\frac{q_{i}q_{j}}{R_{ij}}+\underset{i\in A}{\sum }\underset{j\in B}{\sum }k_{i}k_{j}\left({Ce}^{-\alpha R_{ij}}-\frac{A}{R_{ij}^{6}}\right)$$ 1

This interaction potential combined a standard van der Waals (vdW) term, *E*_*vdW*_^*HS*^,^30^ with a novel electrostatic component (*E*_*elec*_^*HS*^) that used separate charges to model the σ-framework and π-electron cloud of each arene. This was based on a physical picture of planar aromatic systems consisting of a positively charged σ-framework sandwiched between π-electron clouds.^31–33^ The former is represented by positive charges, q_σ_, at the nuclear positions, while the π-electrons are described by a pair of negative charges, q_π_, located a distance ±δ from each atom that is part of the π-system (see Figure 1).

The values of q_σ_ and q_π_ are determined from simple quantum mechanical (QM) computations (see SI) while δ = 0.47 Å was set by requiring that the charges representing benzene result in a molecular quadrupole moment that matches experiment. Charges for benzene and pyridine are shown in Figure 1. In contrast to recent claims,^34^ the application of eq 1 is not dependent upon experimental quadrupole moments; after a simple QM computation on the monomers to determine q_σ_ and q_π_, eq 1 can be applied to any planar aromatic systems comprising H, C, N, and O.^35^ Hunter and Sanders showed^28^ that the locations of energy minima on the resulting interaction potential surfaces match X-ray crystallographic data for a variety of stacked systems and this potential was used extensively over the next decade.^29,36–39^ The electrostatic component was also incorporated into the XED force-field,^40^ which is widely used today.

The simplicity of eq 1 enabled Hunter and Sanders^28^ to extract a “Set of Rules” for the expected geometry and interaction energy of parallel and T-shaped dimers. Although these rules are based on electrostatic arguments, none involve quadrupole moments. The first three concern interactions of aromatic hydrocarbons and include the widespread view that π–π repulsion dominates in the sandwich configuration while π-σ attraction is most important in parallel-displaced and T-shaped configurations. The latter three concern heteroatom and substituent effects and are based on how these groups change the σ- and π-charges of each atom. Although these Rules were based on results from the potential (eq 1), these are distinct and can lead to disparate predictions (*vide infra*).

In the context of the Hunter-Sanders Rules, whether an atom is π-electron-rich or poor is based on the magnitude of the computed q_π_ value. For instance, the *N* of pyridine is π-electron-rich (q_π_ = −0.52, see Figure 1) while C_4_ is π-electron-poor (q_π_ = −0.48). This is different from more modern notions of π-electron-rich and -poor rings, which seem to be based on (a misinterpretation of)^41–44^ molecular electrostatic potentials. For example, while hexafluorobenzene is often offered as a prototypical π-deficient arene, in the context of the Hunter-Sanders Rules the carbons in hexafluorobenzene are π-electron-rich (q_π_ = −0.54). Similarly, while *s*-triazine is considered π-electron-deficient in the parlance of our times, within the Hunter-Sanders Rules the carbon atoms are π-electron-poor (q_π_ = −0.47) and the Ns π-electron-rich (q_π_ = −0.53). The total π-electron charge is (of course) exactly −6, so *s*-triazine itself is neither π-electron-rich nor poor.

Things became more muddled in 2001, when Hunter et al.^22^ published an influential review on aromatic interactions that included a simplified description of the original work from Hunter and Sanders.^28^ Rather than being based on the properties of individual atoms, as in the original model, the emphasis was on the “π-electron system.” More critically, many aromatic interactions were described in terms of molecular quadrupole moments, despite the establishment years earlier that low-order multipole expansions provide inaccurate estimates of the electrostatic component of stacking interactions.^45^ Hunter’s review also introduced an intuitive picture of substituent effects in stacking interactions that quickly took hold. In this model, π-electron-withdrawing substituents (e.g., CN, NO_2_) enhance stacking interactions by removing π-electron density from the ring and relieving some of the π–π repulsion; π-electron-donating substituents (e.g., OH, OMe) hinder stacking through the opposite effect. While this picture has antecedents in the Rules from the original 1990 Hunter-Sanders paper,^28^ it is more accurately described as Hunter’s substituent model; however, it is widely described as the “Hunter-Sanders model.”

Since 2001, “Hunter-Sanders model” has been used to refer to the original Hunter-Sanders potential (eq 1),^28^ the Rules,^28^ the quadrupole-based model from Hunter et al.,^22^ and Hunter’s substituent model,^22^ despite stark difference among these. Most broadly, it is used to describe qualitative models of stacking interactions cast in terms of overall π-electron densities or molecular quadrupole moments.^46^ Some authors are careful to distinguish among these. For instance, Popelier et al.^47^ used “Pictorial Hunter-Sanders model” to denote qualitative models based on molecular quadrupoles and reserved “Hunter-Sanders model” for eq 1. Similarly, Grimme^48^ referred to the quadrupole-based model from Hunter et al.^22^ as “Hunter’s Model.” Other authors are decidedly less precise. For example, in a groundbreaking paper on stacking interactions (*vide infra*), Carter-Fenk and Herbert^49^ at various times used “Hunter-Sanders model” to refer to molecular quadrupole-based models,^22^ the Hunter-Sanders Rules,^28^ and what turns out to be an incomplete version of eq 1.^28^

Many discussions of the “Hunter-Sanders model” have focused on substituent effects on model stacked dimers.^42,50–52^ In 2003, Sinnokrot and Sherrill published^53^ high-accuracy *ab initio* data showing that both electron-donating and withdrawing substituents stabilize the benzene sandwich dimer, seemingly in disagreement with the “Hunter-Sanders Rules.” The following year,^54^ they described these data as disagreeing with the “Hunter-Sanders model,” in apparent reference to the substituent model from Hunter et al.^22^ This seems have been a watershed moment, and “Hunter-Sanders” quickly came to be used synonymously with this π-resonance-based model.^22^ Subsequent computational work^55–61^ continued to chip away at the “Hunter-Sanders” picture of substituent effects. In 2008, Wheeler and Houk^62^ showed that direct interactions between substituents and the other ring, originally proposed by Rashkin and Waters,^63^ were the dominant effect; substituent-induced changes in the π-electron system were unimportant. Our subsequent work^42,43,51,64^ provided additional evidence for the importance of direct interactions, culminating in what we call the Local, Direct Interaction Model^51^ in which substituent effects are due primarily to the direct interaction of each substituent with the proximal vertex of the other ring. More recent computational^64,65^ and experimental^66–71^ studies have confirmed the main tenets of this model.^51^

Remarkably, despite decades of discussion^43,49,53,54,56–58,61,65,69,71,72^ of apparent shortcomings of the “Hunter-Sanders model” in capturing substituent effects in stacking interactions, to our knowledge the Hunter-Sanders potential (eq 1) has never been tested for such systems.^73^ This raises the question of whether data from the Hunter-Sanders potential^28^ agrees with accurate *ab initio* results or with the so-called “Hunter-Sanders model.”^22^

Recently, Carter-Fenk and Herbert^49^ revisited the more fundamental question of the origin of parallel-displaced stacking in the benzene dimer. Hunter and Sanders had explained^28^ this in terms of the competition between electrostatic effects, which (in their model) favor displaced geometries, and vdW interactions, which (in their model) are maximally attractive when the rings overlap.^74^ However, in 2020 Carter-Fenk and Herbert^49^ showed computationally that this picture is qualitatively incorrect. There are two key aspects that Hunter and Sanders missed. First, for distances less than 3.9 Å, charge penetration,^75^ which is neglected in point charge-based models,^76^ leads to a net favorable electrostatic interaction even in the sandwich configuration.^56,77,78^ Consequently, the electrostatic component of the interaction varies little across horizontal displacements at a constant vertical separation. Second, accurately computed vdW potentials exhibit a persistent saddle-point at zero lateral displacement for many stacked dimers. In other words, Carter-Fenk and Herbert showed^49^ that it is vdW interactions, not electrostatic repulsion, that drive the lateral displacement in parallel stacked dimers. This was explained in terms of local maxima in the electron density located above each nucleus, which lead to sharply rising exchange repulsion in geometries in which the nuclei are eclipsed. In an LCAO framework, these ‘lumps’ in the electron density arise from the occupied p_*z*_ orbital on each carbon atom. In other words, Carter-Fenk and Herbert showed^49^ that it is the steric repulsion of overlapping occupied π-orbitals that gives rise to parallel-displaced stacking.

The work of Carter-Fenk and Herbert^49,79^ was based on energies computed at constant vertical separations. Cabaleiro-Lago et al.^80^ showed that if the contributions to the interaction energy are instead evaluated along a minimum energy path (MEP), the electrostatic component is most favorable at parallel displaced geometries while the vdW contribution is at a local maximum, seemingly at odds with the picture painted by Carter-Fenk and Herbert.^49,79^ This was also apparent in the earlier data from Zarić et al.^81^ In 2025, Herbert et al.^46^ offered a more nuanced description of parallel-displaced stacking in the benzene dimer in which vdW interactions control the lateral displacement while electrostatic effects are responsible for pulling the two interacting rings closer at displaced geometries.^79^

In the process of dispensing with the conceptual underpinnings of the Hunter-Sanders explanation of parallel-displaced stacking, Carter-Fenk and Herbert also claimed to show that the Hunter-Sanders potential^28^ fails to predict parallel displaced stacking in the anthracene dimer,^49^ C_6_H_6_···C_6_F_6_ dimer,^49^ and C_6_H_6_···C_96_H_24_ dimer.^79^ However, Carter-Fenk and Herbert^49,79^ consistently describe the potential from ref 28. (eq 1) as consisting of point-charge-based electrostatics combined with dispersion. In other words, these claims were based on calculations that excluded the repulsive term in eq 1.

Carter-Fenk and Herbert^49^ ultimately developed the empirical potential in eq 2 to “replace eq 1”:^82^

$$\begin{array}{c} E_{int}^{CFH}=E_{pauli}^{CFH}+E_{disp}^{aiD3}=\underset{i\in A}{\sum }\underset{j\in B}{\sum }A_{ij}\left(R_{ij}\right)e^{-\alpha_{ij}R_{ij}^{2}}-\underset{i\in A}{\sum }\underset{j\in B}{\sum }\left[f_{6}\left(\beta_{ij}R_{ij}\right)\frac{C_{ij}}{R_{ij}^{6}}+f_{8}\left(\beta_{ij}R_{ij}\right)\frac{D_{ij}}{R_{ij}^{8}}\right] \end{array}$$ 2

In this alternative potential, the Born-Mayer repulsive term^83,84^ is replaced with a spherical Gaussian overlap model, the R^6^ dispersion component is updated with a damped dispersion model (aiD3),^85,86^ and the electrostatic term is removed.^82^ Herbert et al.^34,49,79,82^ have consistently stated that eq 2 describes the conformational behavior of a range of parallel and T-shaped dimers while the original Hunter-Sanders potential does so “only for the benzene dimer” where it works “by accident.”^87^

In 2021, Herbert et al.^82^ published an Account that includes a discussion of the Hunter-Sanders model in which they now present the correct form of eq 1. They applied this to the parallel and T-shaped benzene dimers, showing that it fails in the latter case because the potential is too flat along lateral displacements! Consequently, they offered this damning assessment: “*In three decades of discussing the Hunter–Sanders model, it is unclear that anyone ever bothered to check its behavior for the T-shaped isomer*.” They also applied eq 1 to the C_6_H_6_···C_96_H_24_ dimer, finding that it fails by incorrectly predicting that there is an energy minimum at the eclipsed geometry. However, interaction energies for this system computed using the correct form of eq 1 are indistinguishable from their previously published data^79^ that lacked the repulsive term. Something was clearly amiss and Herbert et al.^88^ recently acknowledged that these data were based on a flawed implementation of eq 1; correctly implemented, it provides qualitatively correct potentials for these systems.

Below, we first reassess the performance of the Hunter-Sanders potential (eq 1)^28^ for parallel and T-shaped dimers of aromatic hydrocarbons and compare this to the proposed replacement^49,82^ from Carter-Fenk and Herbert (eq 2). Next, we test whether the Hunter-Sanders potential can reliably describe heteroatom effects in parallel-displaced stacking interactions. Finally, we consider the ability of eq 1 to capture substituent effects in parallel-stacked benzene dimers. The results are in stark contrast to what one would expect based on the modern literature.

## 2 Computational Methods

The implementation of eqs 1 and 2 using AaronTools^89^ is described in Supporting Information (SI). We note that eq 2 is not defined for vertical separations below 3.5 Å; consequently, while we show data below this cutoff in the two-dimensional plots, when taking slices at constant vertical separations we follow ref 49 and use *z* = 3.5 Å for distances below this value. Similarly, eq 2 was never intended to be used quantitatively.^49,79^ As such, we focus on the shape of the resulting potential. Data from these potentials are compared to interaction energies computed at the SAPT2+3/def2-TZVPD^90–92^ level of theory, which provides accurate total interaction energies (see SI Figure S1) as a combination of electrostatic (Elec), exchange repulsion (Exch), induction (Ind), and dispersion (Disp) effects. SAPT computations were performed using Psi4,^93^ with structure manipulation and input file generation done using AaronTools.^89^ See SI for details.

## 3 Results and Discussion

### Aromatic Hydrocarbons

Interaction energies for the parallel stacked benzene dimer are shown in Figure 2 as a function of horizontal (*x*) and vertical (*z*) displacements (see Figure 1) computed using SAPT along with eqs 1 and 2. Figure 3 shows these interaction energies along the MEP of the corresponding two-dimensional surface and at a fixed vertical separation of 3.5 Å. The parallel stacked benzene dimer potential surface is characterized by two equivalent energy minima at lateral displacements of ±1.7 Å separated by a saddle point corresponding to the sandwich configuration roughly 1 kcal/mol higher in energy. The vertical separation at the saddle point is 3.9 Å while at the minima this distance is reduced to 3.5 Å.

**Figure 2:**
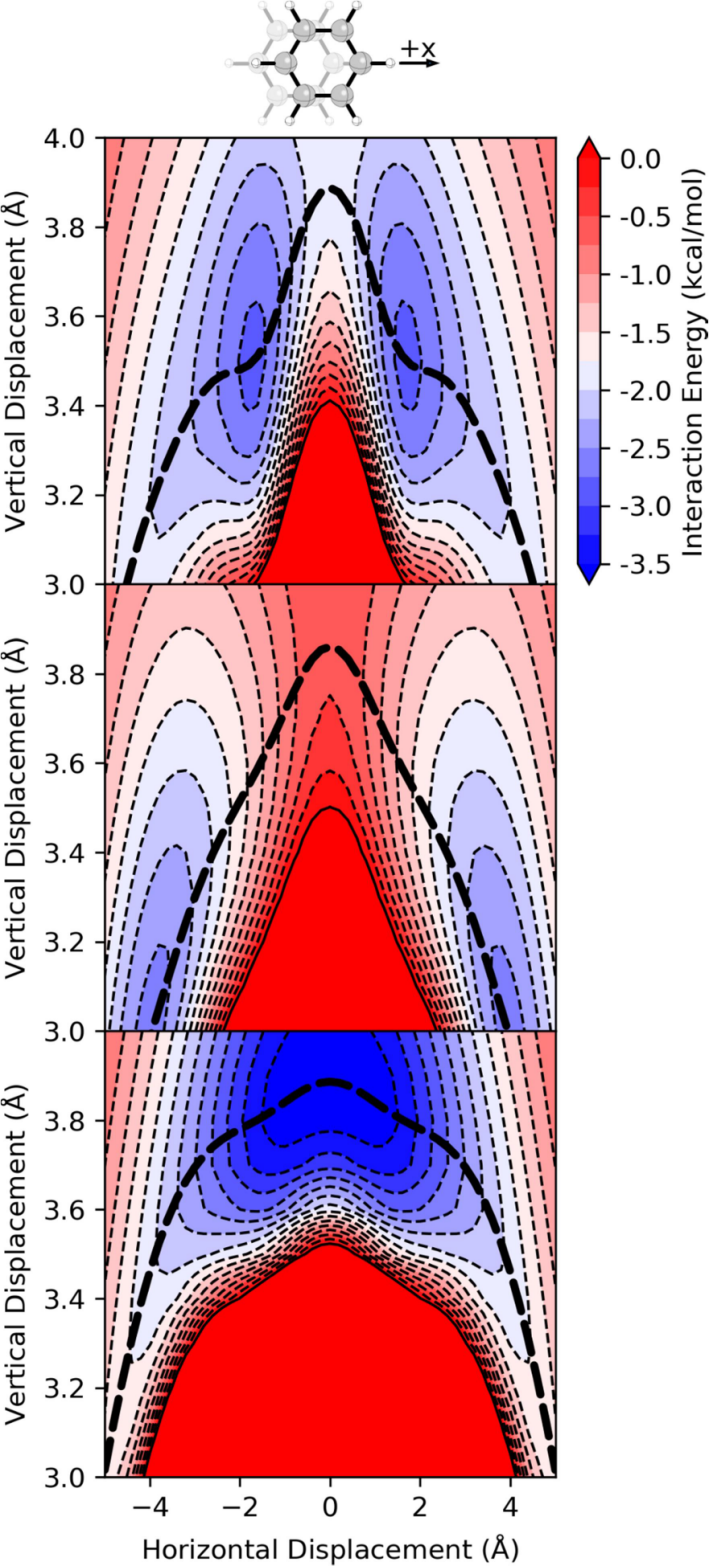
Interaction energies for the parallel stacked benzene dimer from (top) SAPT, (middle) the Hunter-Sanders potential (eq 1), and (bottom) the Carter-Fenk-Herbert potential (eq 2). The bold dashed lines show the MEPs along each potential. Horizontal displacement of *x* = 0 corresponds to the sandwich dimer (see Figure 1).

**Figure 3:**
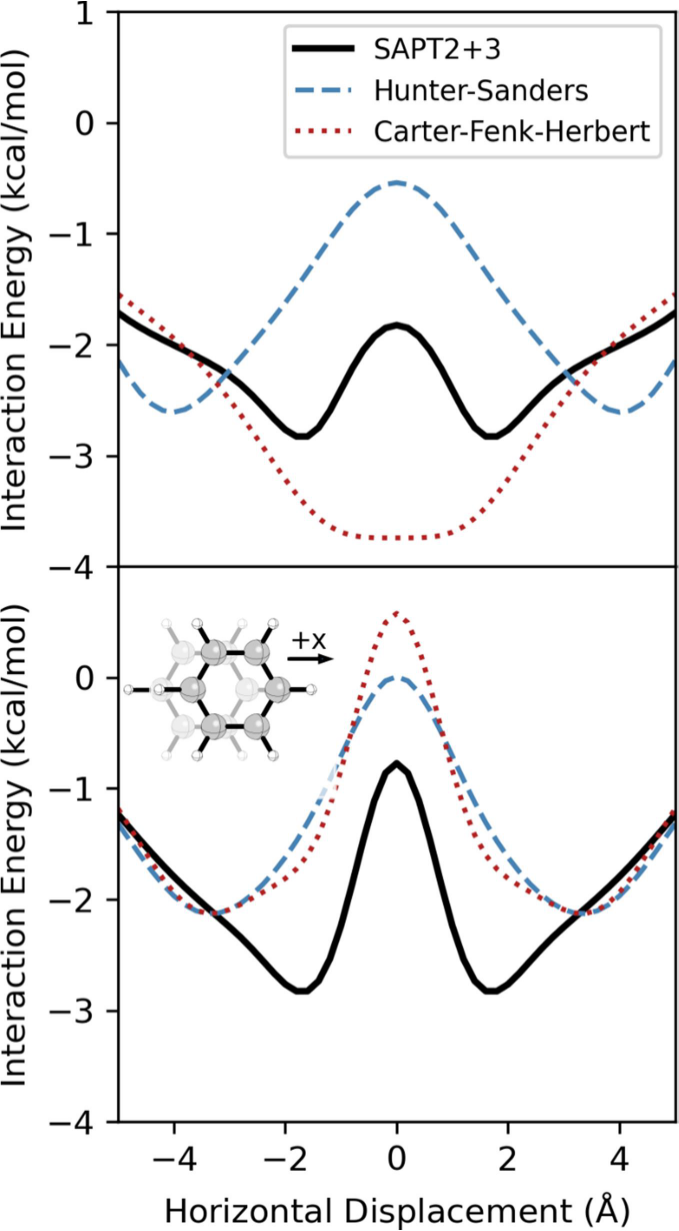
Interaction energies from SAPT, the Hunter-Sanders potential (eq 1), and the Carter-Fenk-Herbert potential (eq 2) for the parallel stacked benzene dimer for (top) the MEP and (bottom) *z* = 3.5 Å.

The Hunter-Sanders potential (eq 1) reproduces the basic shape of this potential and correctly predicts the vertical separation of the sandwich dimer. However, there are notable shortcomings. First, eq 1 overestimates the horizontal displacement of the minima and underestimates the vertical separation at these geometries. Furthermore, while the depth of the energy minima agrees with the SAPT results, eq 1 severely underestimates the favorability of the sandwich dimer. The energy curve for *z* = 3.5 Å from eq 1 (Figure 3) is slightly more accurate but still overestimates the lateral displacement at the energy minima and the barrier height.

The vdW and electrostatic contributions to the interaction energy from SAPT and eq 1 are plotted in Figure 4. The vdW component of eq 1 underestimates the repulsive component, predicting favorable interactions as close as *z* = 3 for some lateral displacements. More critically, while there is a small ridge in *E*_*vdW*_^*HS*^ corresponding to the eclipsed configuration, this ridge does not extend sufficiently far along the *z*-coordinate. Consequently, even though scans through *E*_*vdW*_^*HS*^ for *z* < 3.5 Å exhibit a saddle point at *x* = 0, this critical ‘bump’ is missing for larger vertical separations. The result is that the vdW component of eq 1 lacks the double-well topography that Herbert et al.^46^ have shown is a persistent feature of stacked aromatic systems. At the same time, the electrostatic component of eq 1 is qualitatively incorrect; it is unfavorable for all z-values for |*x*| < 3 Å while exact electrostatics indicates an attractive potential across all lateral displacements for *z* < 3.9 Å. As discussed by Herbert et al.^34,46,49,79,82,87^ and others,^80^ this is due to the neglect of charge-penetration effects in eq 1.

**Figure 4:**
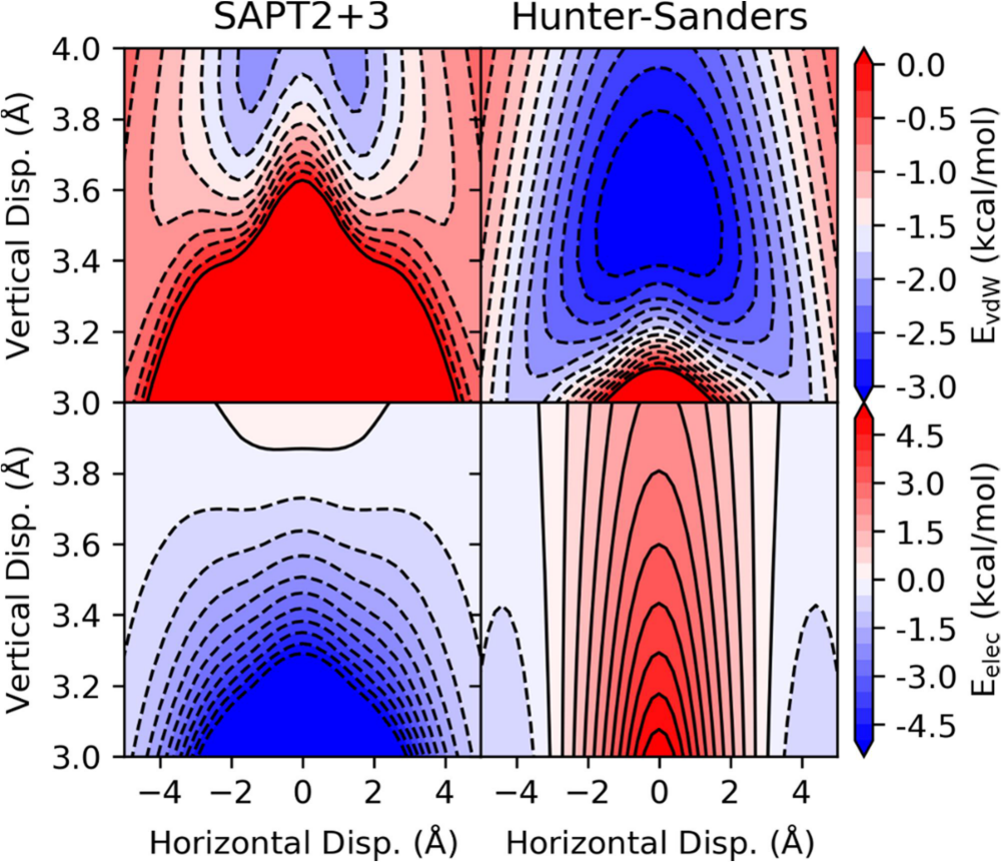
vdW (top) and electrostatic (bottom) contribution to the interaction energy of the parallel benzene dimer from SAPT (left) and the Hunter-Sanders potential (right). For SAPT, *E*_vdW_ = *E*_exch_ + *E*_disp_.

Given the errors in the vdW and electrostatic components of eq 1, it is remarkable that the total interaction energy provides a qualitatively correct picture for the parallel benzene dimer. In this sense, there is a kernal of truth in the claim from Gray and Herbert^87^ that the Hunter-Sanders potential only gets the benzene dimer correct “by accident.” Hunter and Sanders^28^ explicitly noted that they did not expect the individual terms in eq 1 to be quantitatively correct; however, they implicitly assumed that the these terms were qualitatively correct, as these formed the basis of their eponymous Rules. This assumption turned out to be unfounded, and ultimately led to the incorrect conclusion that parallel-displaced stacking is driven by electrostatic effects.^34,49,79,82^

The repulsive ridge in the vdW component of eq 1 for eclipsed configurations does not extend to large enough vertical separations, leading Hunter and Sanders^28^ to miss the critical role played by vdW interactions in driving parallel-displaced stacking in the benzene dimer. The empirical model proposed by Carter-Fenk and Herbert^49^ (eq 2) as a replacement^82^ for eq 1 appears to suffer from the same defect, although to a lesser degree (see Figure 2, and note that eq 2 consists solely of a vdW component). While the repulsive region in the potential from eq 2 more closely matches the SAPT vdW data, the ridge at *x* = 0 disappears around *z* = 3.8 Å. Consequently, even though eq 2 gives a double-well potential energy curve for *z* = 3.5 Å (for which it seems to have been parametrized; see SI Sec. S3), the overall potential energy surface features a single energy minimum at (*x*, *z*) = (0, 3.9 Å). This can be seen in the MEP for eq 2 in Figure 3, which shows a single, broad energy minimum. In other words, while the Hunter-Sanders potential (eq 1) predicts the qualitatively correct behavior for the parallel benzene dimer for the wrong reason, the proposed replacement^82^ from Carter-Fenk and Herbert (eq 2)^49^ does not predict the correct behavior. That the vdW components of both eqs 1 and 2 do not exhibit the correct double-well shape is unsurprising given the simplicity of the repulsive components of both potentials.

Interaction potentials from SAPT2 + 3 and eqs 1 and 2 are plotted for the T-shaped benzene dimer in Figures 5 and 6. The interaction energy surface produced by eq 1 is remarkably accurate. This includes the overall shape of the potential and strong preference for a C_2v_-symmetric geometry. The only deficiencies are that the location of the minimum is off by 0.1 Å and the energy well is slightly too shallow. In terms of one-dimensional interaction energy curves, for both the MEP and a slice at *z* = 5.0 Å (see Figure 6) the shape of the potential from eq 1 matches the SAPT data, albeit with features that are slightly muted. Moreover, unlike the parallel case, the individual vdW and electrostatic components from eq 1 (see SI Figure S2) are in reasonable agreement with SAPT data; the electrostatic component underestimates the favorability for small vertical separations due to the lack of charge penetration, but this is compensated by a slightly reduced repulsive term from the vdW component. Overall, the Hunter-Sanders potential (eq 1) predicts the correct behavior for the T-shaped benzene dimer for the correct reason.

**Figure 5:**
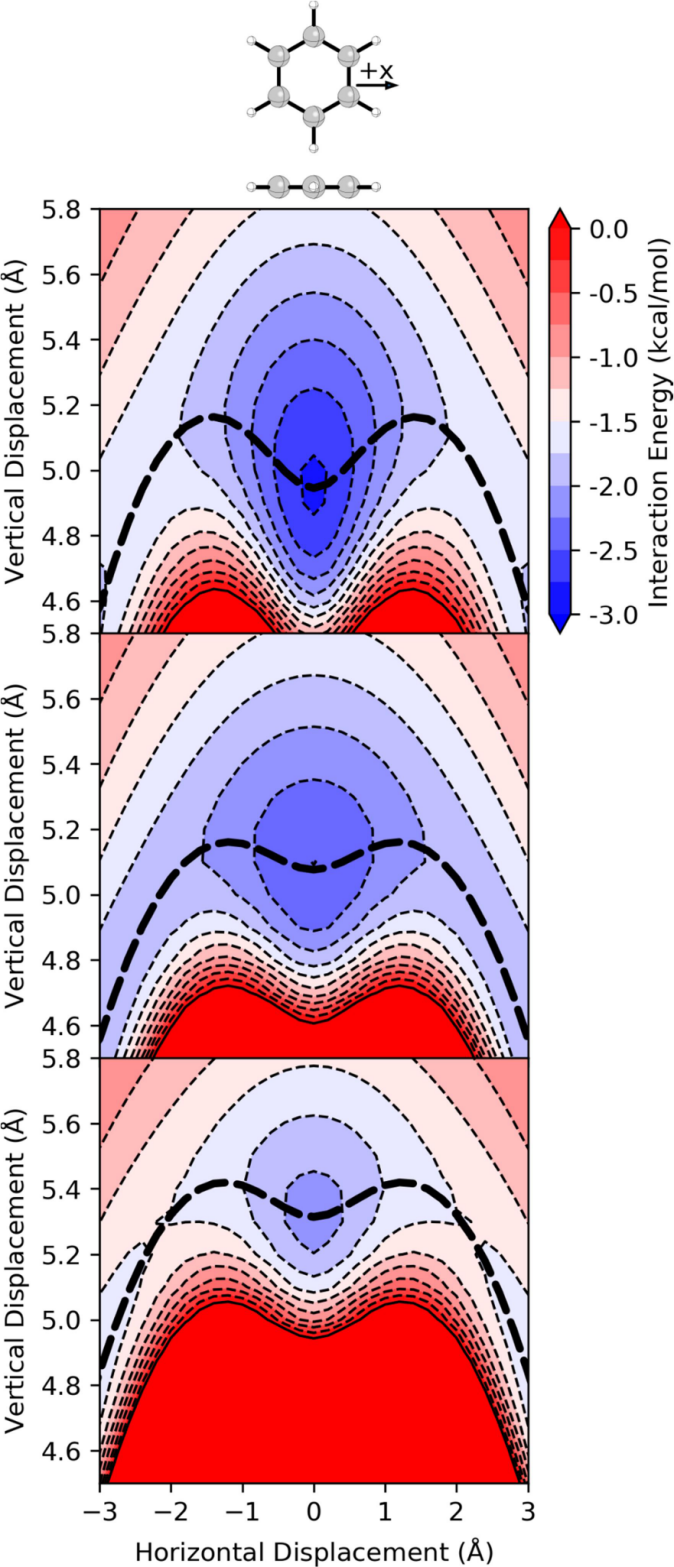
Interaction energies for the T-shaped benzene dimer from (top) SAPT, (middle) the Hunter-Sanders potential (eq 1), and (bottom) the Carter-Fenk-Herbert potential (eq 2). The bold dashed line indicates the MEP along each surface.

**Figure 6:**
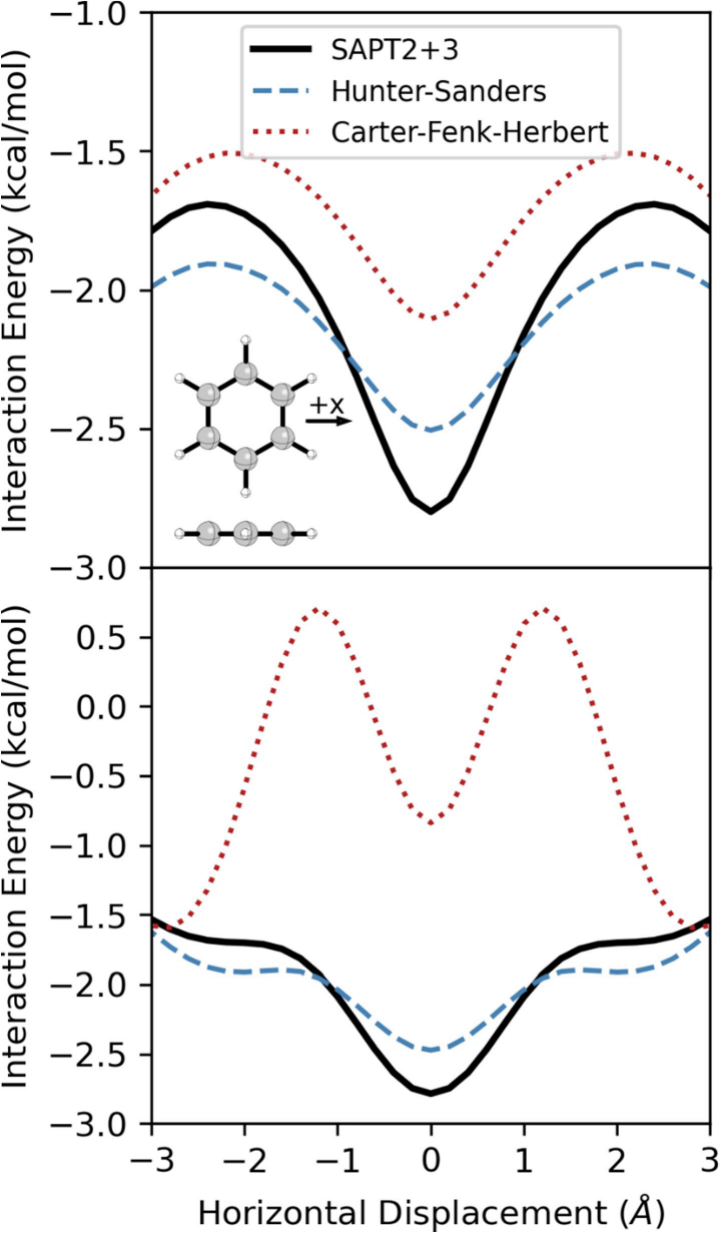
Interaction energies from SAPT, the Hunter-Sanders potential (eq 1), and the Carter-Fenk-Herbert potential (eq 2) for the T-shaped benzene dimer for (top) the corresponding MEP and (bottom) *z* = 5.0 Å.

The Carter-Fenk-Herbert potential (eq 2) fares better for the T-shaped benzene dimer than it does for the parallel case; however, in terms of the shape of the potential energy curve along the MEP it offers no improvement over eq 1. Moreover, the scan at *z* = 5.0 Å drastically overestimates the repulsive interaction around *x* = ± 1.3 Å. The reason is that even though eq 2 is a decent match for the vdW component from SAPT, it fails to account for the role of electrostatic effects in softening these repulsive walls.

The situation for the parallel-stacked anthracene dimer is similar (see SI Figures S3); data from eq 1 conflict with the conclusions from Carter-Fenk and Herbert,^49^ which were based on calculations that excluded the repulsive term. While the Hunter-Sanders potential underestimates the strength of the interaction and the magnitude of the features on the potential, it captures the favorability of parallel-displaced stacking. At the same time, the Carter-Fenk-Herbert potential fails to predict displacement along the long axis of anthracene, instead predicting a single energy minimum. For the perpendicular (T-shaped) anthracene dimer, the Carter-Fenk-Herbert potential (eq 2) performs well, with the energy along the MEP closely paralleling that from DFT (See SI Figure S4). However, as seen for the benzene T-shaped dimer, energies for *z* = 5 Å overestimate the oscillations in the potential and fail to predict the differences in energy among the local minima. The Hunter-Sanders potential (eq 1) provides the correct overall topography, but with interaction energies systematically too weak; similarly, the features on the surface are decidedly muted.

For the dimer of benzene with C_96_H_24_, the interaction potential from eq 1 is qualitatively correct, but the magnitude of the interaction is again underestimated and the features on the potential muted. The shape of the energy curve along the MEP from the Carter-Fenk-Herbert potential (eq 2) offers no improvement, while the energy at *z* = 3.5 Å is remarkably close to that from SAPT (See SI Figure S5).

### Heteroatom Effects

Next, we turn to heteroatoms, which can induce significant changes in the shape and relative energies of the parallel-displaced energy minima in parallel stacked dimers. Herbert et al.^46^ showed that even accurately computed vdW potentials are unable to capture many salient features of the interaction potential for stacked heterocyclic dimers, including the relative energy of the minima, because these effects are dominated by electrostatics. Consequently, one would not expect the Carter-Fenk-Herbert potential (eq 2, which neglects electrostatic effects) to describe the interactions of stacked heterocycles (see SI Sec. S4). As a result, we focus here on the ability of the potential from Hunter and Sanders (eq 1) to capture heteroatom effects in parallel-displaced stacking, which was a major focus of their original work.^28^

SAPT2+3 data are plotted in Figure 7 for the benzene-pyridine dimer. The introduction of nitrogen induces an asymmetry in the energy surface resulting in the minimum with the pyridine *N* displaced away from the benzene (+*x*) favored by 0.8 kcal/mol over the other minimum. It is instructive to compare the energy along the corresponding MEPs for benzene-pyridine and benzene dimers (see Figure 7). In the sandwich configuration (*x* = 0), introducing N enhances the stacking interaction by 0.5 kcal/mol.^94^ This enhancement extends uniformly for + *x* displacements out past the energy minimum to *x* = 2 Å. Going the other way, the introduction of *N* hinders stacking. The overall effect of introducing a single *N* atom is a lowering of the interaction energy surface combined with a ‘tilting’ of the energy surface toward + *x* displacements. These effects are primarily electrostatic in origin and are counteracted to a small extent by vdW interactions that are slightly more favorable for *x* ≤ 0 in the pyridine-benzene dimer compared to the benzene dimer (See SI Figure S7).

**Figure 7:**
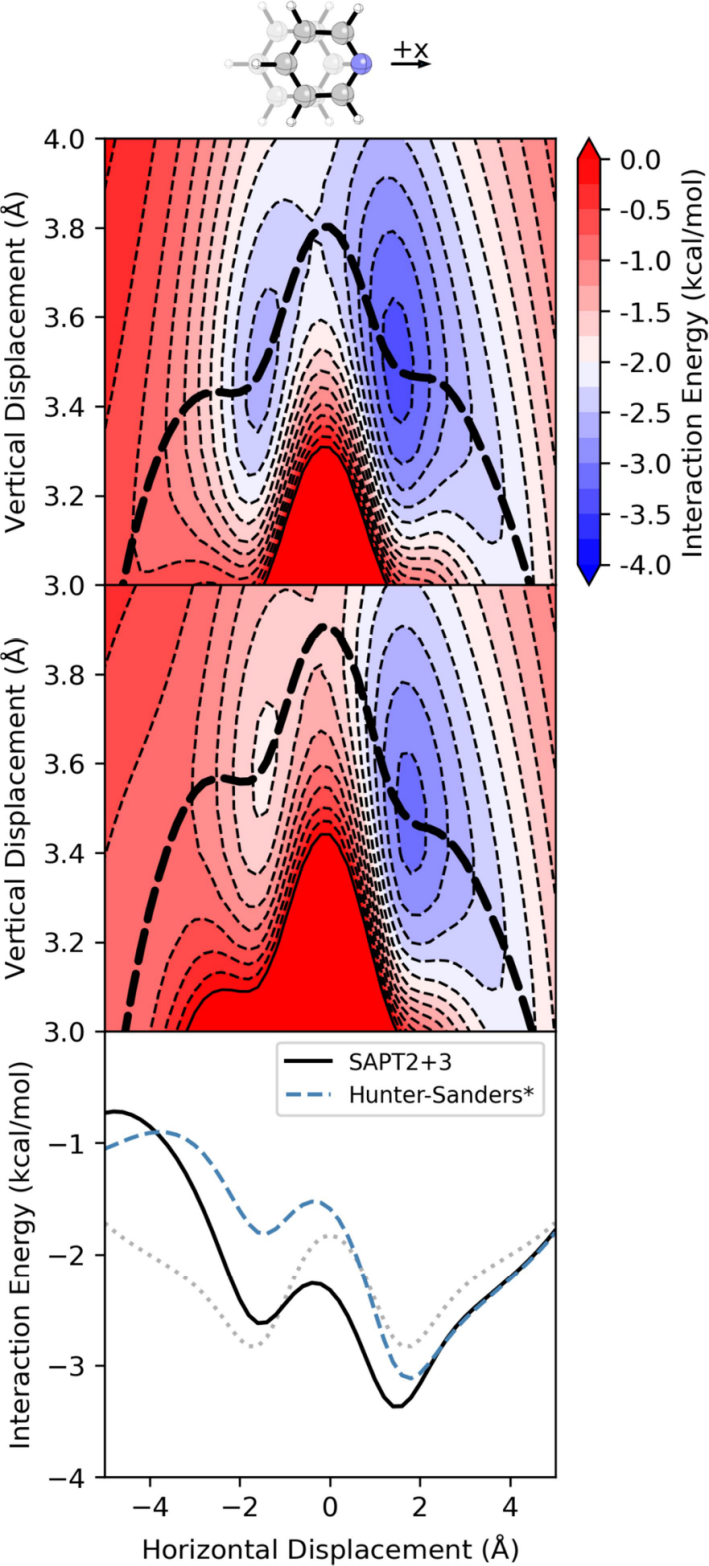
Interaction energy for the parallel-stacked pyridine-benzene dimer from (top) SAPT and (middle) eq 3. The dashed line is the MEP across the corresponding potential. (bottom) Interaction energy along the corresponding MEP computed using SAPT and eq 3 (Hunter-Sanders*). The dotted light gray curve is the SAPT MEP for the benzene dimer, for reference.

Data from the direct application of eq 1 to the pyridine-benzene dimer inherits the defects from the benzene dimer (see SI Figure S6); the energy minima correspond to |*x*| and *z* values that are too large and too small, respectively and the barrier separating the two minima is exaggerated. To isolate the ability of the Hunter-Sanders model to account for heteroatom effects from the underlying benzene dimer interaction potential, we consider interaction energies for heterocyclic dimers relative to the benzene dimer. To plot a more intuitive quantity, we compare SAPT interaction energies for the heterocyclic systems with interaction energies in which the difference in energy between the heterocyclic system and benzene dimer computed using eq 1 is added to the benzene dimer interaction energy from SAPT:

$$E_{int}^{HS*}\left(X\cdots C_{6}H_{6}\right)=E_{int}^{SAPT}\left(C_{6}H_{6}\cdots C_{6}H_{6}\right)+\left[E_{int}^{HS}\left(X\cdots C_{6}H_{6}\right)-E_{int}^{HS}\left(C_{6}H_{6}\cdots C_{6}H_{6}\right)\right]$$ 3

Interaction energies computed using eq 3 are plotted in Figure 7 for the benzene-pyridine dimer. The Hunter-Sanders model reliably captures the impact of the *N*-heteroatom on the symmetry of the potential, preferentially stabilizing geometries displaced along the + *x* direction; however, it overestimates the tilting effect, resulting in significant errors along -*x* displacements. Comparing the energies along the corresponding MEPs, eq 3 fails to predict the enhanced stacking due to *N* in the sandwich configuration (*x* = 0); however, it accurately captures the impact of *N* beyond the + *x* minimum. Overall, eq 3 gets the direction of the energetic bias correct but overestimates the impact of the heteroatom. Considering the contributions of electrostatic and vdW interactions (see SI Figure S7), the Hunter-Sanders point charge model reliably describes the electrostatic impact of the *N*; however, the vdW component fails to capture the stabilizing effect of replacing CH with N for *x* ≤ 0, instead predicting a slightly less favorable interaction.

It should be noted that the direction of tilting of the interaction energy surface for the pyridine-benzene dimer is also correctly predicted by the Hunter and Sanders Rules.^28^ As noted above, for pyridine the *N* atom is π-electron rich and C_4_ π-electron-poor. The Rules correctly predict that the most favorable stacking will arise if the π-electron-deficient C_4_ is placed over the face of the benzene (+*x* displacement) and that stacking will be weaker if the π-electron-rich N is over the other ring (-*x* displacement).

We also considered homodimers of pyridine and *s*-triazine in aligned and antialigned configurations. Energies along the MEPs from SAPT and eq 3 are plotted in Figure 8 (2D potential energy surfaces are available in SI). For the pyridine-dimer, the Hunter-Sanders potential qualitatively captures the impact of the heteroatoms but overestimates the effect and misses the lowering of the interaction energy due to the presence of the *N*-atoms. Consequently, the MEP from eq 3 parallels the SAPT data for the aligned configuration but underestimates the strength of stacking across all horizontal displacements. For the antialigned dimer, eq 3 predicts an energy minimum along + *x* displacements in reasonable agreement with SAPT but predicts only a shoulder rather than an energy minimum along -*x* displacements. This is consistent with the data for the benzene-pyridine dimer—eq 1 falters for geometries in which an *N* atom is placed over the face of another ring. Predictions from the Hunter-Sanders Rules are also in accord with the overall picture for the pyridine dimer, including the fact that the antialigned dimer stacks more favorably than the aligned case and that the favored direction of displacement for the antialigned dimer shifts the two *N* atoms away from the other ring.

**Figure 8:**
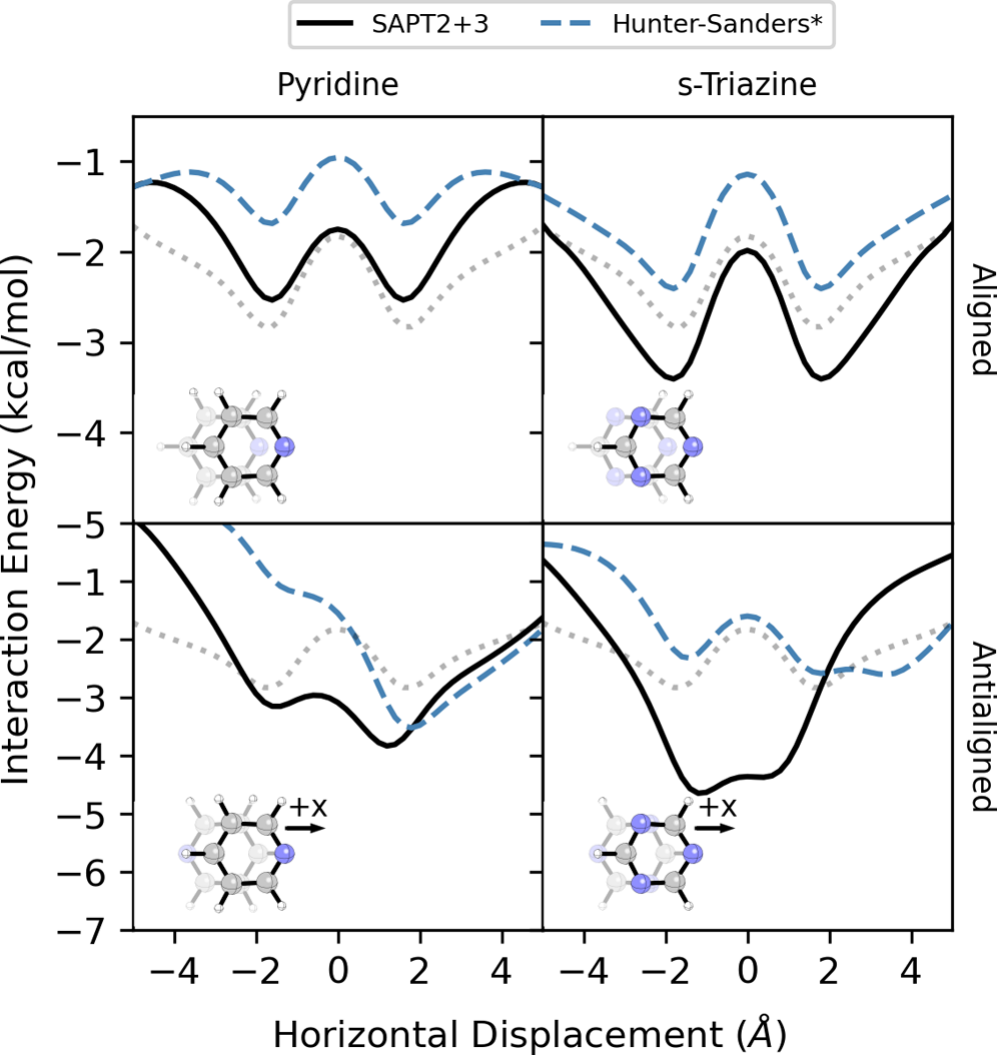
Energies along the MEP for aligned and antialigned parallel-stacked dimers of pyridine and s-triazine computed using SAPT and eq 3 (Hunter-Sanders*). Dotted light gray curves show the SAPT MEP for the benzene dimer for comparison.

Predictably, given that eq 3 struggles to accurately capture the impact of displacements that place *N* over another ring, it inaccurately describes the *s*-triazine dimer in either aligned or antialigned configurations. In the aligned dimer, eq 3 erroneously predicts hindered stacking across all horizontal displacements. For the antialigned dimer, eq 3 also predicts weaker stacking than the benzene dimer across most distances, whereas SAPT indicates strongly enhanced stacking for *x* < 2 Å.

### Substituent Effects

Finally, we consider the effect of substituents on stacking interactions in the benzene dimer, which have been the focus of much of the criticism of the “Hunter-Sanders model” over the last 20 years.^42,50,51,53–62,64–71,95^ We first consider a diverse group of 17 monosubstituted benzene sandwich dimers (see SI Table S1). As with heteroatom effects, the Carter-Fenk-Herbert potential (eq 2) is not expected to capture substituent effects, for which electrostatic interactions play a pivotal role^42,43,50,51,53–58,61,62,64,65,77,95,96^ (see SI Figure S22). As such, we focus on the ability of the Hunter-Sanders potential (eq 1) to capture these effects.

Interaction energies, relative to the unsubstituted case (X = H), computed using eq 1 are plotted in Figure 9 versus data from SAPT at the optimized vertical separations from ref 51. (see SI for data). There is a strong correlation (r^2^ = 0.91) and a root mean squared error (RMSE) of only 0.2 kcal/mol. That is, even though the Hunter-Sanders potential underestimates the favorability of stacking in the benzene sandwich dimer (−0.2 vs −1.8 kcal/mol), it accurately captures the effect of substituents on this interaction energy. Critically, this includes the fact that all substituents enhance stacking, relative to the benzene dimer. For example, eq 1 correctly predicts that the dimers of toluene, aniline, phenol, and anisole with benzene stack more strongly than the benzene dimer. For decades, it has been claimed^42,50,51,53–62,64–71,95^ that the “Hunter-Sanders model” is unable to reproduce this behavior. Those claims apply to the 2001 model of Hunter et al.^22^ and the Hunter-Sanders Rules; the Hunter-Sanders potential^28^ accurately captures this effect. The primary reason is that eq 1 is inclusive of both electrostatic and dispersion effects and accounts for both σ- and π-polarization.

**Figure 9:**
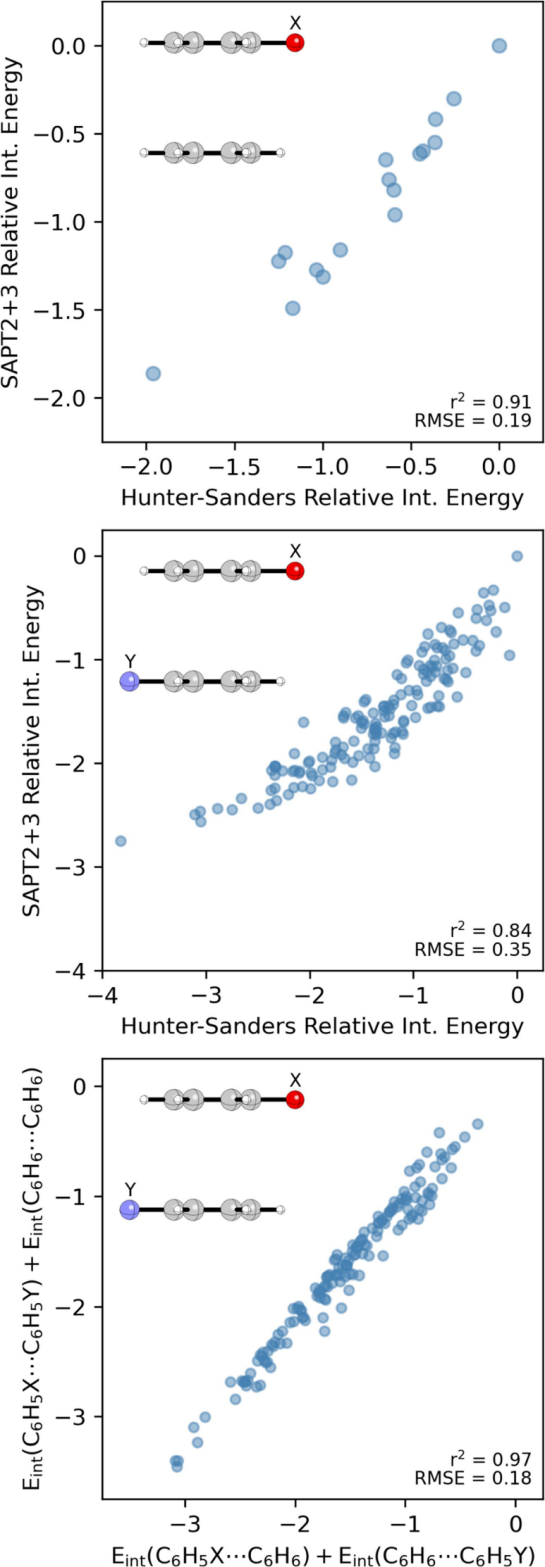
Correlation of SAPT relative interaction energies (kcal/mol) with predictions from eq 1 for (top) monosubstituted sandwich dimers and (middle) mixed C_6_H_5_X···C_6_H_5_Y dimers. (bottom) Correlation of interaction energies from eq 1 for mixed C_6_H_5_X···C_6_H_5_Y dimers vs the sum of individual substituent effects.

We also considered C_6_H_5_X···C_6_H_5_Y sandwich dimers with the substituents oriented in opposite directions. Interaction energies for 153 mixed dimers of monosubstituted benzenes, relative to the unsubstituted case (X, Y = H), predicted using eq 1 are compared with SAPT results in Figure 9. There is a more modest correlation (r^2^ = 0.84) and larger RMSE (0.4 kcal/mol) in this case. The largest errors occur for the strongest interacting dimers, whose favorability is overestimated by the Hunter-Sanders potential. For instance, eq 1 predicts a relative interaction energy of −3.8 kcal/mol for X, Y = NO_2_, whereas SAPT gives a value of −2.7 kcal/mol. Regardless, the Hunter-Sanders potential provides qualitatively correct predictions of substituent effects in these more challenging dimers. Moreover, the vdW and electrostatic components, relative to the benzene dimer, are relatively accurate, particularly in the latter case (See SI Figure S12). In other words, eq 1 appears to capture substituent effects in the benzene sandwich dimer for the right reasons.

A hallmark of Hunter’s 2001 substituent effect model^22^ is that the nature of the substituent on one ring determines the effect of a substituent on the other ring.^97^ In contrast to this, our Local, Direct Interaction Model^51^ predicts that the effect of X and Y will be independent as long as the substituents are not in each other’s local environment (i.e., as long as direct X···Y interactions are negligible). Data for the C_6_H_5_X···C_6_H_5_Y dimers predicted using eq 1 are plotted against the sum of interaction energies from C_6_H_5_X···C_6_H_6_ and C_6_H_6_···C_6_H_5_Y from the same model in Figure 9. There is a very strong correlation (r^2^ = 0.97) with a RMSE of only 0.2 kcal/mol. In other words, the Hunter-Sanders potential (eq 1)^28^ predicts substituent effects that are in accord with our Local, Direct Interaction Model^51^ but in contrast to those from the “Hunter-Sander model!”^22^ Apparently, the importance of direct interactions^62,63^ were present all along in the Hunter-Sanders potential;^28^ they appear to have just been overlooked in the formulation of the Rules and subsequent substituent model from Hunter et al.^22^

While the Hunter-Sanders potential^28^ reliably captures the impact of substituents in the benzene sandwich dimer, it falls short when considering substituent effects as a function of lateral displacements. We considered parallel displaced C_6_H_5_X···C_6_H_6_ dimers in which the substituted ring is displaced along the C_1_–C_4_ axis (*e.g*. see Figure 10). Arnstein and Sherrill^55^ showed that the geometry with the substituent over the other ring (-*x* displacements) is energetically preferred for many substituents systems. This can be seen in the SAPT data for the benzonitrile-benzene dimer in Figure 10. The introduction of CN strengthens the interaction for most horizontal displacements; however, the effect is strongest along the -x direction. The result is an asymmetric double-well potential in which the minimum along the -*x* direction lies 0.8 kcal/mol lower than the minimum displaced along the + *x* direction. The SAPT data indicate that the -*x* minimum is primarily stabilized by vdW interactions (and slightly destabilized by electrostatic effects) while stabilizing electrostatic effects are dominant in the +*x* minimum­(see SI Figure S13).

**Figure 10:**
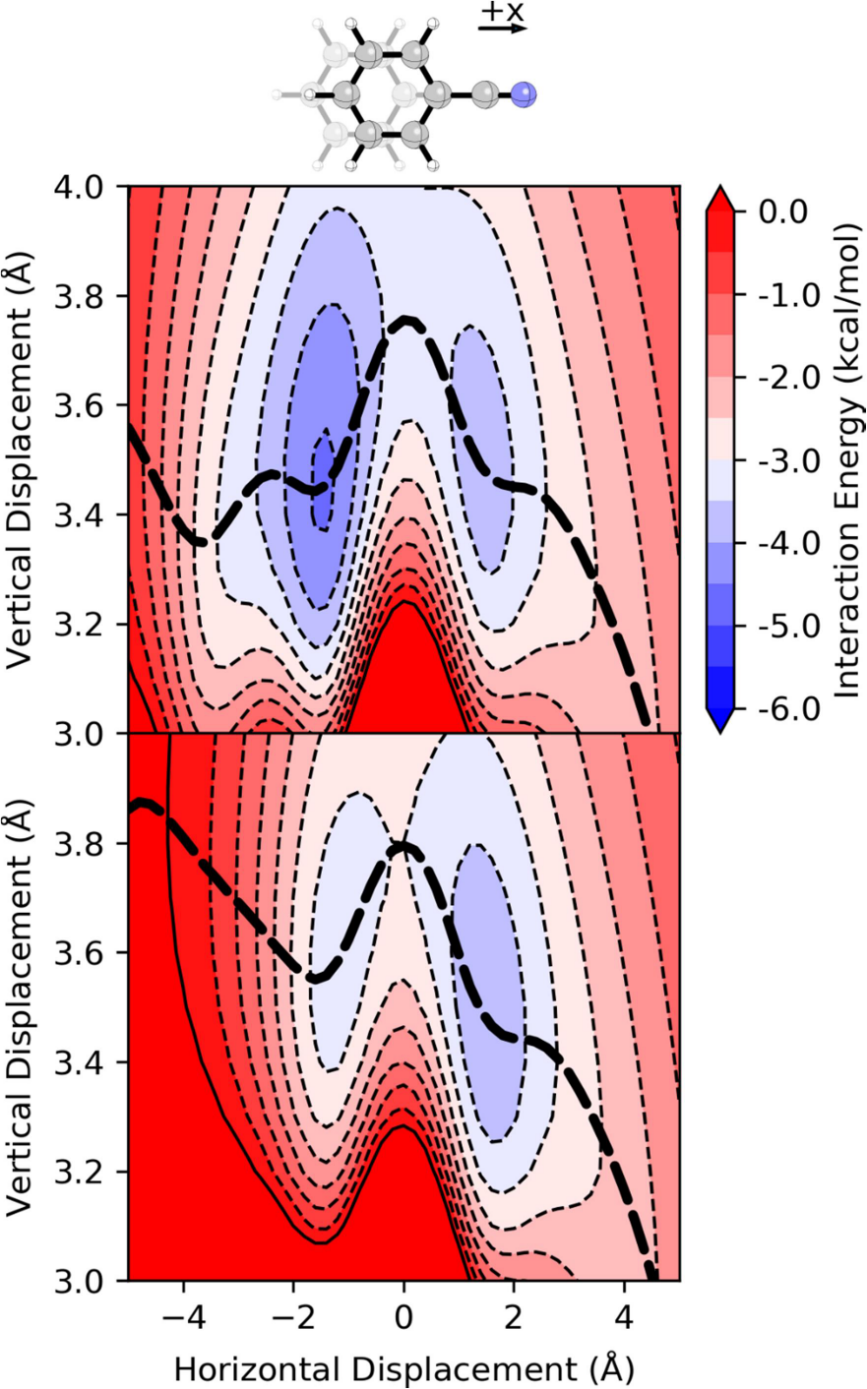
Interaction energy for the parallel-stacked benzonitrile-benzene dimer from (top) SAPT and (bottom) eq 3 (Hunter-Sanders*). The dashed line is the MEP across the corresponding potential.

As with the heterocycle dimers, direct application of eq 1 to substituted benzene dimers inherits the flaws of the benzene dimer potential. Instead, interaction energies from eq 3 are plotted in Figure 10 for the benzonitrile-benzene dimer. While eq 3 predicts an asymmetric interaction potential energy surface, the surface is tilted in the wrong direction. The result is the minimum corresponding to + *x* displacement is favored by 0.6 kcal/mol over the geometry with the nitrile group positioned over the face of the rother ring. Closer examination of the individual components reveals that the Hunter-Sanders potential includes too steep of an electrostatic penalty and slightly insufficient enhancement of the vdW component for -*x* displacements (see SI Figure S13).

The data in Figure 10 are not anomalous. Energies along the MEPs from SAPT and eq 3 are plotted in Figure 11 for six monosubstituted benzene dimers (2-D potential energy surfaces are available in SI Figures S14–18). In each case, the Hunter-Sanders potential correctly captures the substituent effect for + *x* displacements, relative to the unsubstituted case. The data are in near quantitative agreement for CN, CH_3_, and CCH. However, the energy curves for *x* < 0 are qualitatively incorrect for all but the toluene-benzene dimer. Even in that case, eq 3 misses the second energy minimum at *x* = −3 Å, which arises from a CH/π intraction^98^ of the methyl group with the unsubstituted ring. The net result is that even when layered on top of accurate interaction energies for the benzene dimer, the Hunter-Sanders potential is unable to predict the preferred direction of parallel displacement (-x) for these monosubstituted benzene dimers.

**Figure 11:**
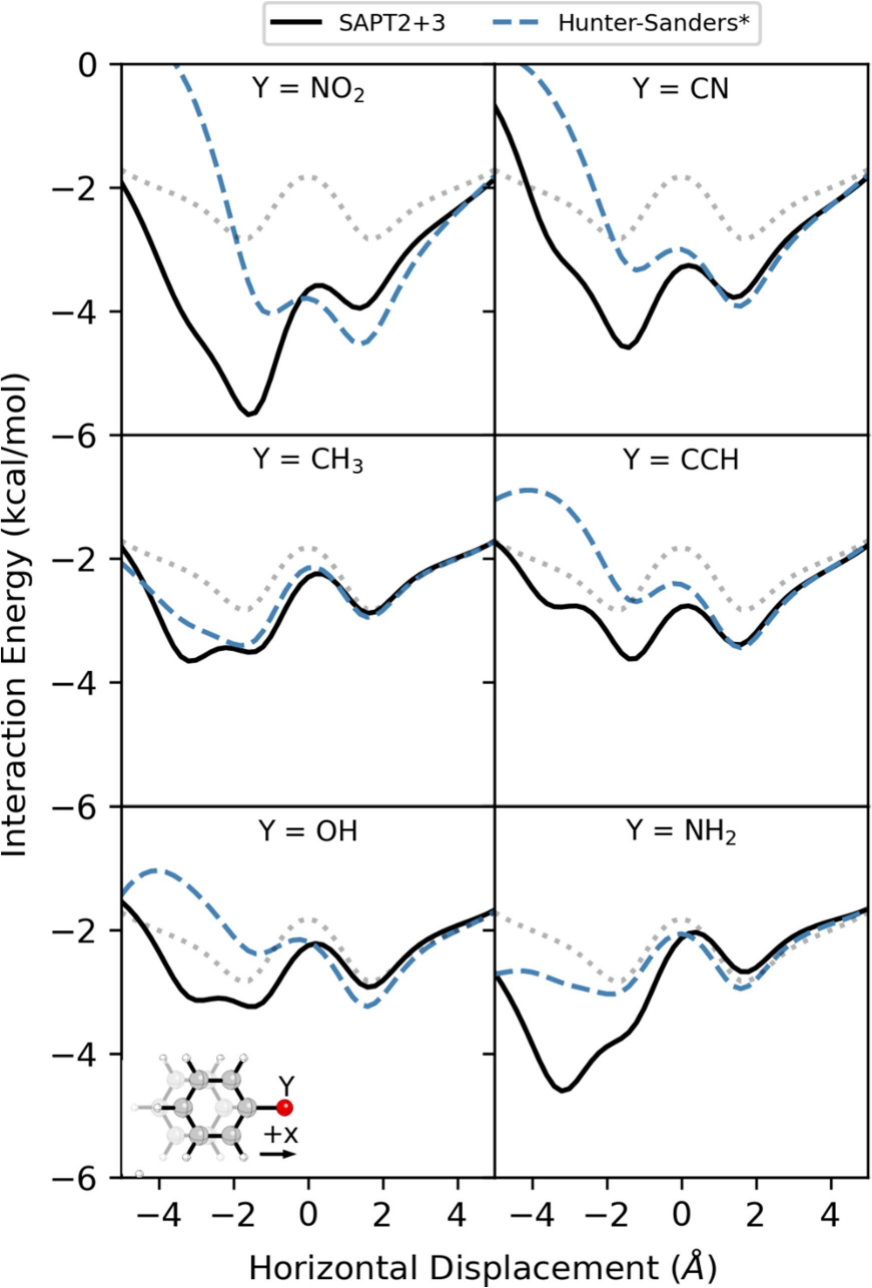
Interaction energies along the corresponding MEP for parallel-stacked C_6_H_5_Y···C_6_H_6_ dimers computed using SAPT (black) and eq 3 (Hunter-Sanders*, blue). The dotted light gray curve is the SAPT MEP for the benzene dimer, for reference.

## 4 Concluding Remarks

All models are wrong, but some are useful.^99^ The utility of a model flows from its predictive and interpretive power. Chemistry is a field built on models (chemical bonds, molecular orbitals, transition state theory, *etc*.) and all of these, if you dig down deep enough, are wrong. They are, however, useful because they allow us to understand complex chemical systems and make often reliable qualitative and even quantitative predictions without resorting to expensive computations.

Stacking interactions are no exception; chemists rely on qualitative and quantitative models to make sense of these ubiquitous noncovalent interactions and predict their behavior. The “Hunter-Sanders model” is foundational to many chemists’ understanding of stacking interactions. Unfortunately, over the last 35 years the meaning of “Hunter-Sanders model” has come untethered from the contents of the paper by Hunter and Sanders.^28^ Consequently, this phrase is widely used to describe an array of models couched in terms of π-electron densities or molecular quadrupole moments that do not appear in ref 28. The original Hunter-Sanders model,^28^ either in the form of the interaction potential in eq 1 or the eponymous ‘Rules,’ was based on charges associated with individual atoms.

The conflation of these models is detrimental, because the most discussed shortcomings^34,42,49–52,79,80,82^ of the “Hunter-Sanders model” do not apply to the original potential from Hunter and Sanders.^28^ For example, despite repeated claims that the “Hunter-Sanders model” fails to describe substituent effects in the benzene sandwich dimer, the data in Figure 9 show that eq 1 performs well. In terms of substituent effects in parallel-displaced stacked dimers, the Hunter-Sanders potential captures the impact of substituents for geometries in which the substituent moves away from the other ring but fails to capture these effects for dimers in which the substituent is positioned over the other ring. Similarly, the Hunter-Sanders potential accurately captures the impact of heteroatoms on parallel-displaced geometries in which the heteroatom is displaced away from the other ring but overestimates the destabilizing effects in parallel displaced dimers in which heteroatoms are located over the face of the other ring. Finally, the qualitative Hunter-Sanders Rules^28^ appear to provide correct guidance in terms of the impact of heteroatoms on the strength of stacking, although they falter when applied to substituent effects.^53,63^

In 2020, in the process of dispensing with the widespread view that parallel-displaced stacking is driven by electrostatic effects, as in the original work of Hunter and Sanders,^28^ Carter-Fenk and Herbert^49^ proposed an empirical potential (eq 2) to replace the original Hunter-Sanders potential (eq 1). Unfortunately, this proposed replacement does not appear to offer improvement over the original potential. Moreover, eq 2 is unable to predict parallel-displaced stacking for several model systems, including the benzene dimer!

The point of this work is not to champion the Hunter-Sanders model^28^ or to justify the use of eq 1 in modern applications. The primary goal is to correct the scientific record. Testing the limits of the models on which we depend to understand and predict stacking interactions is important. However, it is contrary to scientific progress to summarily dismiss a model as a “failure,” however flawed parts of it are, based on data computed using an incomplete or incorrect incarnation of that model. Many aspects of the Hunter-Sanders model^28^ are flawed; however, others appear to provide qualitatively correct predictions. It is important to make that distinction to aid the development of more robust conceptual models.

## Supplementary Material













## Notes

Computed energies and SAPT components (XLSX)
Cartesian coordinates (XYZ)
Cartesian coordinates for systems in Figure 9 (XYZ)
Computed charges (TXT)
Computed charges for systems in Figure 9 (TXT)
Additional figures, tables, and computational details (PDF)

## References

[1] Meyer E. A., Castellano R. K., Diederich F. (2003). Interactions with Aromatic Rings
in Chemical and Biological Recognition. Angew.
Chem., Int. Ed..

[2] Schneider H. J. (2009). Binding
mechanisms in supramolecular complexes. Angew.
Chem., Int. Ed. Engl..

[3] Salonen L. M., Ellermann M., Diederich F. (2011). Aromatic rings in chemical and biological
recognition: energetics and structures. Angew.
Chem., Int. Ed. Engl..

[4] Bhat V., Callaway C. P., Risko C. (2023). Computational
Approaches for Organic
Semiconductors: From Chemical and Physical Understanding to Predicting
New Materials. Chem. Rev..

[5] Anthony J. E. (2006). Functionalized
acenes and heteroacenes for organic electronics. Chem. Rev..

[6] Mullin W. J., Müller P., Schaefer A. J., Guzman E., Wheeler S. E., Thomas S. W. (2022). Crystal engineering of heterocyclic
arylene­(ethynylene) oligomers through programmed aromatic stacking. Journal of Materials Chemistry C.

[7] Wang C., Li Z. (2017). Molecular conformation
and packing: their critical roles in the emission
performance of mechanochromic fluorescence materials. Materials Chemistry Frontiers.

[8] Neel A. J., Hilton M. J., Sigman M. S., Toste F. D. (2017). Exploiting non-covalent
pi interactions for catalyst design. Nature.

[9] Wheeler S.
E., Seguin T. J., Guan Y., Doney A. C. (2016). Noncovalent Interactions
in Organocatalysis and the Prospect of Computational Catalyst Design. Acc. Chem. Res..

[10] Maji R., Mallojjala S. C., Wheeler S. E. (2023). Electrostatic Interactions in Asymmetric
Organocatalysis. Acc. Chem. Res..

[11] Lee W. G., Gallardo-Macias R., Frey K. M., Spasov K. A., Bollini M., Anderson K. S., Jorgensen W. L. (2013). Picomolar inhibitors of HIV reverse
transcriptase featuring bicyclic replacement of a cyanovinylphenyl
group. J. Am. Chem. Soc..

[12] Kim J. T., Hamilton A. D., Bailey C. M., Domaoal R. A., Wang L., Anderson K. S., Jorgensen W. L. (2006). FEP-guided
selection of bicyclic
heterocycles in lead optimization for non-nucleoside inhibitors of
HIV-1 reverse transcriptase. J. Am. Chem. Soc..

[13] Clark M. P., Ledeboer M. W., Davies I., Byrn R. A., Jones S. M., Perola E., Tsai A., Jacobs M., Nti-Addae K., Bandarage U. K. (2014). Discovery of a Novel, First-in-Class, Orally
Bioavailable Azaindole Inhibitor (VX-787) of Influenza PB2. J. Med. Chem..

[14] Murray J., Giannetti A. M., Steffek M., Gibbons P., Hearn B. R., Cohen F., Tam C., Pozniak C., Bravo B., Lewcock J. (2014). Tailoring
small molecules for an allosteric site on
procaspase-6. ChemMedChem..

[15] Togo T., Tram L., Denton L. G., ElHilali-Pollard X., Gu J., Jiang J., Liu C., Zhao Y., Zhao Y., Zheng Y. (2023). Systematic Study of Heteroarene Stacking Using a Congeneric
Set of Molecular Glues for Procaspase-6. J.
Med. Chem..

[16] Ertl P., Altmann E., Racine S., Lewis R. (2022). Ring replacement
recommender:
Ring modifications for improving biological activity. Eur. J. Med. Chem..

[17] Tyagarajan S., Lowden C. T., Peng Z., Dykstra K. D., Sherer E. C., Krska S. W. (2015). Heterocyclic Regioisomer Enumeration
(HREMS): A Cheminformatics
Design Tool. J. Chem. Inf Model.

[18] Bootsma A. N., Doney A. C., Wheeler S. E. (2019). Predicting the Strength of Stacking
Interactions between Heterocycles and Aromatic Amino Acid Side Chains. J. Am. Chem. Soc..

[19] Bootsma A. N., Wheeler S. E. (2018). Stacking Interactions
of Heterocyclic Drug Fragments
with Protein Amide Backbones. ChemMedChem..

[20] Bootsma A. N., Wheeler S. E. (2019). Converting SMILES to Stacking Interaction Energies. J. Chem. Inf Model.

[21] Conner A. V., Kim L. M., Fagan P. A., Harding D. P., Wheeler S. E. (2025). Stacking
Interactions of Druglike Heterocycles with Nucleobases. J. Chem. Inf Model.

[22] Hunter C. A., Lawson K. R., Perkins J., Urch C. J. (2001). Aromatic Interactions. J. Chem.
Soc., Perkin Trans..

[23] Sinnokrot M. O., Valeev E. F., Sherrill C. D. (2002). Estimates of the Ab Initio Limit
for π–π Interactions: The Benzene Dimer. J. Am. Chem. Soc..

[24] Czernek J., Brus J. (2024). Revisiting the Most Stable Structures of the Benzene Dimer. Int. J. Mol. Sci..

[25] Schnell M., Erlekam U., Bunker P. R., von Helden G., Grabow J. U., Meijer G., van der Avoird A. (2013). Structure
of the benzene dimer--governed by dynamics. Angew. Chem., Int. Ed. Engl..

[26] Tsuzuki S., Honda K., Uchimaru T., Mikami M. (2004). High-level ab initio
computations of structures and interaction energies of naphthalene
dimers: origin of attraction and its directionality. J. Chem. Phys..

[27] Podeszwa R., Szalewicz K. (2008). Physical origins
of interactions in dimers of polycyclic
aromatic hydrocarbons. Phys. Chem. Chem. Phys..

[28] Hunter C.
A., Sanders J. K. M. (1990). The
Nature of π-π Interactions. J. Am.
Chem. Soc..

[29] Hunter C. A., Singh J., Thornton J. M. (1991). Pi-pi interactions: the geometry
and energetics of phenylalanine-phenylalanine interactions in proteins. J. Mol. Biol..

[30] Caillet J., Claverie P. (1975). Theoretical evaluations
of the intermolecular interaction
energy of a crystal: application to the analysis of crystal geometry. Acta Crystallogr., Sect. A.

[31] Kekulé A. (1865). Sur la constitution
des substances aromatiques. Bull. Soc. Chim.
Fr..

[32] Armit J. W., Robinson R. (1925). CCXI.—Polynuclear
heterocyclic aromatic types.
Part II. Some anhydronium bases. J. Chem. Soc.,
Trans..

[33] Lonsdale K. (1929). The structure
of the benzene ring in C6­(CH3)­6.. Proc. R. Soc.
London, Ser. Ar.

[34] Herbert J. M. (2021). Neat, Simple,
and Wrong: Debunking Electrostatic Fallacies Regarding Noncovalent
Interactions. J. Phys. Chem. A.

[35] The van der Waal component of Eq. 1 was only parameterized for these elements in Ref 30.

[36] Hunter C. A. (1993). Sequence-dependent
DNA structure. The role of base stacking interactions. J. Mol. Biol..

[37] Hunter C. A., Lu X.-J., Kapteijn G. M., van Koten G. (1995). Influence
of fluorine on aromatic interactions. Journal
of the Chemical Society, Faraday Transactions.

[38] Hunter C. A., Lu X. J. (1997). DNA base-stacking
interactions: a comparison of theoretical calculations
with oligonucleotide X-ray crystal structures. J. Mol. Biol..

[39] Packer M. J., Dauncey M. P., Hunter C. A. (2000). Sequence-dependent DNA structure:
dinucleotide conformational maps. J. Mol. Biol..

[40] Vinter J. G. (1994). Extended
electron distributions applied to the molecular mechanics of some
intermolecular interactions. J. Comput. Aided
Mol. Des.

[41] Wheeler S. E., Houk K. N. (2009). Through-Space Effects of Substituents Dominate Molecular
Electrostatic Potentials of Substituted Arenes. J. Chem. Theory Comput..

[42] Wheeler S. E. (2013). Understanding
Substituent Effects in Non-Covalent Interactions Involving Aromatic
Rings. Acc. Chem. Res..

[43] Wheeler S. E., Bloom J. W. (2014). Toward a more complete
understanding of noncovalent
interactions involving aromatic rings. J. Phys.
Chem. A.

[44] Wheeler S. E., Bloom J. W. (2014). Anion-pi interactions and positive electrostatic potentials
of N-heterocycles arise from the positions of the nuclei, not changes
in the pi-electron distribution. Chem. Commun.
(Camb).

[45] Price S. L., Stone A. J. (1987). The electrostatic interactions in van der Waals complexes
involving aromatic molecules. J. Chem. Phys..

[46] Schramm B., Gray M., Herbert J. M. (2025). Substituent
and Heteroatom Effects
on pi-pi Interactions: Evidence That Parallel-Displaced pi-Stacking
is Not Driven by Quadrupolar Electrostatics. J. Am. Chem. Soc..

[47] Triestram L., Falcioni F., Popelier P. L. A. (2023). Interacting
Quantum Atoms and Multipolar
Electrostatic Study of XH···pi Interactions. *ACS*. Omega.

[48] Grimme S. (2008). Do Special
Noncovalent π-π Stacking Interactions Really Exist?. Angew. Chem., Int. Ed..

[49] Carter-Fenk K., Herbert J. M. (2020). Electrostatics does not dictate the slip-stacked arrangement
of aromatic pi-pi interactions. Chem. Sci..

[50] Raju R. K., Bloom J. W. G., An Y., Wheeler S. E. (2011). Substituent Effects
in Non-Covalent Interactions with Aromatic Rings: Insights from Computational
Chemistry. ChemPhysChem.

[51] Wheeler S. E. (2011). Local Nature
of Substituent Effects in Stacking Interactions. J. Am. Chem. Soc..

[52] Fagnani, D. E. ; Sotuyo, A. ; Castellano, R. K. π-π Interactions. In Comprehensive Supramolecular Chemistry II, Atwood, J. L. , Ed.; Vol. 1; Elsevier, 2013; pp 121–148.

[53] Sinnokrot M. O., Sherrill C. D. (2003). Unexpected Substituent
Effects in Face-to-Face π-Stacking
Interactions. J. Phys. Chem. A.

[54] Sinnokrot M. O., Sherrill C. D. (2004). Substituent Effects
in π-π Interactions:
Sandwich and T-Shaped Configurations. J. Am.
Chem. Soc..

[55] Arnstein S.
A., Sherrill C. D. (2008). Substituent
Effects in Parallel-Sisplaced π-π
Interactions. Phys. Chem. Chem. Phys..

[56] Hohenstein E. G., Duan J., Sherrill C. D. (2011). Origin
of the Surprising Enhancement
of Electrostatic Energies by Electron-Donating Substituents in Substituted
Benzene Sandwich Dimers. J. Am. Chem. Soc..

[57] Ringer A. L., Sherrill C. D. (2009). Substituent Effects
in Sandwich Configurations of Multiply
Substituted Benzene Dimers Are Not Solely Governed By Electrostatic
Control. J. Am. Chem. Soc..

[58] Ringer A. L., Sinnokrot M. O., Lively R. P., Sherrill C. D. (2006). The Effect of Multiple
Substituents on Sandwich and T-Shaped π-π Interactions. Chem. - Eur. J..

[59] Lee E. C., Kim D., Jurecka P., Tarakeshwar P., Hobza P., Kim K. S. (2007). Understanding
of assembly phenomena by aromatic-aromatic interactions: benzene dimer
and the substituted systems. J. Phys. Chem.
A.

[60] Seo J.-I., Kim I., Lee Y. S. (2009). π-π
Interaction Energies in Monosubstituted-Benzene
Dimers in Parallel- and antiparallel-Displaced Conformations. Chem. Phys. Lett..

[61] Watt M., Hardebeck L. K., Kirkpatrick C. C., Lewis M. (2011). Face-to-face arene-arene
binding energies: dominated by dispersion but predicted by electrostatic
and dispersion/polarizability substituent constants. J. Am. Chem. Soc..

[62] Wheeler S. E., Houk K. N. (2008). Substituent Effects
in the Benzene Dimer are Due to
Direct Interactions of the Substituents with the Unsubstituted Benzene. J. Am. Chem. Soc..

[63] Rashkin M. J., Waters M. L. (2002). Unexpected Substituent Effects in Offset π–π
Stacked Interactions in Water. J. Am. Chem.
Soc..

[64] Raju R. K., Bloom J. W. G., Wheeler S. E. (2013). Broad Transferability
of Substituent
Effects in π-Stacking Interactions Provides New Insights into
Their Origin. J. Chem. Theory Comput..

[65] Parrish R. M., Sherrill C. D. (2014). Quantum-mechanical
evaluation of pi-pi versus substituent-pi
interactions in pi stacking: direct evidence for the Wheeler-Houk
picture. J. Am. Chem. Soc..

[66] Snyder S. E., Huang B. S., Chu Y. W., Lin H. S., Carey J. R. (2012). The effects
of substituents on the geometry of pi-pi interactions. Chemistry.

[67] Hwang J., Li P., Carroll W. R., Smith M. D., Pellechia P. J., Shimizu K. D. (2014). Additivity of substituent
effects in aromatic stacking
interactions. J. Am. Chem. Soc..

[68] Harder M., Carnero Corrales M. A., Trapp N., Kuhn B., Diederich F. (2015). Rebek imide
platforms as model systems for the investigation of weak intermolecular
interactions. Chemistry.

[69] Hwang J., Li P., Vik E. C., Karki I., Shimizu K. D. (2019). Study of through-space
substituent−π interactions using N-phenylimide molecular
balances. Organic Chemistry Frontiers.

[70] Burns R. J., Mati I. K., Muchowska K. B., Adam C., Cockroft S. L. (2020). Quantifying
Through-Space Substituent Effects. Angew. Chem.,
Int. Ed. Engl..

[71] Snyder S. E., Huang B. S., Chen Y. T., Lin H. S., Carey J. R. (2012). A simple
chiral recognition system to investigate substituent effects on pi-pi
interactions. Org. Lett..

[72] Sinnokrot M. O., Sherrill C. D. (2006). High-Accuracy Quantum
Mechanical Studies of π-π
Interactions in Benzene Dimers. J. Phys. Chem.
A.

[73] Hunter and Sanders looked at dimers involving benzene, p-diaminobenzene, and p-benzoquinone in various geometries, but only reported the electrostatic contribution to the interaction energy.

[74] It is often stated that Hunter and Sanders described parallel displaced stacking in terms of the competition between electrostatics and dispersion. They did not.

[75] Stone, A. J. The Theory of Intermolecular Forces; Oxford University Press, 1996.

[76] Rackers J.
A., Wang Q., Liu C., Piquemal J. P., Ren P., Ponder J. W. (2017). An optimized charge
penetration model for use with
the AMOEBA force field. Phys. Chem. Chem. Phys..

[77] Sherrill C.
D. (2013). Energy
component analysis of pi interactions. Acc.
Chem. Res..

[78] Ryno S. M., Risko C., Brédas J.-L. (2016). Noncovalent Interactions and Impact
of Charge Penetration Effects in Linear Oligoacene Dimers and Single
Crystals. Chem. Mater..

[79] Carter-Fenk K., Herbert J. M. (2020). Reinterpreting pi-stacking. Phys.
Chem. Chem. Phys..

[80] Cabaleiro-Lago E. M., Rodriguez-Otero J., Vazquez S. A. (2022). Electrostatic penetration effects
stand at the heart of aromatic pi interactions. Phys. Chem. Chem. Phys..

[81] Ninkovic D. B., Blagojevic Filipovic J. P., Hall M. B., Brothers E. N., Zaric S. D. (2020). What Is Special
about Aromatic-Aromatic Interactions?
Significant Attraction at Large Horizontal Displacement. ACS Cent Sci..

[82] Carter-Fenk K., Lao K. U., Herbert J. M. (2021). Predicting and Understanding
Non-Covalent
Interactions Using Novel Forms of Symmetry-Adapted Perturbation Theory. Acc. Chem. Res..

[83] Born M., Mayer J. E. (1932). Zur Gittertheorie
der Ionenkristalle. Zeitschrift fr Physik.

[84] Buckingham R. A. (1938). The classical
equation of state of gaseous helium, neon and argon.. Proc. R. Soc. London, Ser. A.

[85] Lao K. U., Herbert J. M. (2015). Accurate and efficient quantum chemistry calculations
for noncovalent interactions in many-body systems: the XSAPT family
of methods. J. Phys. Chem. A.

[86] Tang K. T., Toennies J. P. (1984). An improved simple
model for the van der Waals potential
based on universal damping functions for the dispersion coefficients. J. Chem. Phys..

[87] Gray M., Herbert J. M. (2023). Origins of Offset-Stacking
in Porous Frameworks. J. Phys. Chem. C.

[88] Carter-Fenk K., Lao K. U., Herbert J. M. (2025). Correction to ″Predicting
and Understanding Noncovalent Interactions Using Novel Forms of Symmetry-Adapted
Perturbation Theory″. Acc. Chem. Res..

[89] Ingman V. M., Schaefer A. J., Andreola L. R., Wheeler S. E. (2020). QChASM: Quantum
chemistry automation and structure manipulation. WIREs Comput. Mol. Sci..

[90] Hohenstein E. G., Sherrill C. D. (2010). Density Fitting of Intramonomer Correlation Effects
in Symmetry-Adapted Perturbation Theory. J.
Chem. Phys..

[91] Hohenstein E. G., Sherrill C. D. (2012). Wavefunction methods
for noncovalent interactions. WIREs Comp. Mol.
Sci..

[92] Weigend F., Ahlrichs R. (2005). Balanced basis sets
of split valence, triple zeta valence
and quadruple zeta valence quality for H to Rn: Design and assessment
of accuracy. Phys. Chem. Chem. Phys..

[93] Turney J. M., Simmonett A. C., Parrish R. M., Hohenstein E. G., Evangelista F. A., Fermann J. T., Mintz B. J., Burns L. A., Wilke J. J., Abrams M. L. (2012). PSI4: an open-source
ab initio electronic structure program. Wires
Comput. Mol. Sci..

[94] Hohenstein E. G., Sherrill C. D. (2009). Effects of Heteroatoms On Aromatic π-π
Interactions: Benzene-Pyridine and Pyridine Dimer. J. Phys. Chem. A.

[95] Wheeler S. E., McNeil A. J., Müller P., Swager T. M., Houk K. N. (2010). Probing
Substituent Effects in Aryl–Aryl Interactions Using Stereoselective
Diels–Alder Cycloadditions. J. Am. Chem.
Soc..

[96] Sherrill, C. D. Computations of Noncovalent pi interactions. In Reviews in Computational Chemistry, Lipkowitz, K. B. ; Cundari, T. R. , Eds.; Vol. 26; Wiley-VCH, 2009; pp 1–38.

[97] Cockroft S. L., Perkins J., Zonta C., Adams H., Spey S. E., Low C. M. R., Vinter J. G., Lawson K. R., Urch C. J., Hunter C. A. (2007). Substituent Effects on Aromatic Stacking Interactions. Org. Biomol. Chem..

[98] Bloom J. W. G., Raju R. K., Wheeler S. E. (2012). Physical Nature of Substituent Effects
in XH/π Interactions. J. Chem. Theory
and Comput..

[99] Box G. E.
P. (1976). Science
and Statistics. Journal of the American Statistical
Association.

