# Supplementary material for: Revisiting the Hunter-Sanders Model for π–π Interactions
Source: J Am Chem Soc. 2025 May 29;147(23):19738–50. doi: 10.1021/jacs.5c03169 (PMC12164356; doi:10.1021/jacs.5c03169)
Supplement: Supplementary file 6 [file ja5c03169_si_006.pdf]

Supporting Information

Revisiting the Hunter-Sanders Model for π-π Interactions

Steven E. Wheeler[*]

Department of Chemistry, University of Georgia, Athens, GA 30602
E-mail: swheele2@uga.edu

Contents pp.

S1. Additional Figures and Table	S2
S2. Implementation of Equations 1 and 2	S21
S3. Origin of the Scaling Parameter in the Carter-Fenk-Herbert vdW Potential	S22
S4. Carter-Fenk-Herbert Potential Applied to Heterocyclic and Substituted Systems	S24
S5. Computational Details	S28
S6. Other Data	S29
**References**	S29

S1. Additional Figures and Table

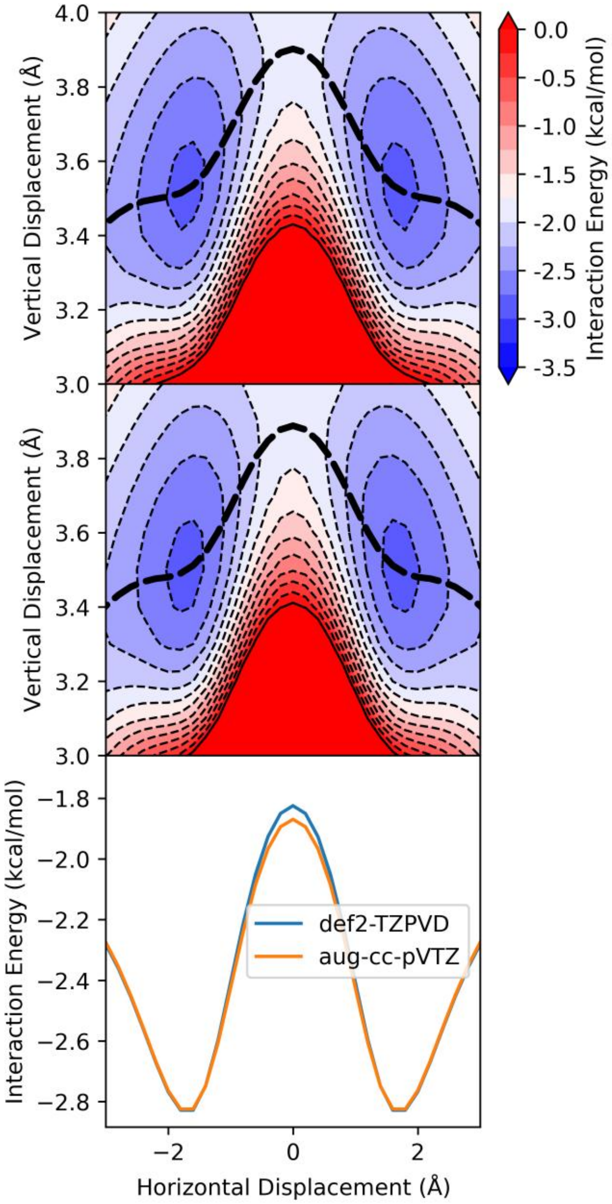

Figure S1. (top) SAPT2+3(δMP2)/aug-cc-pVTZ and (middle) SAPT2+3/def2-TZVPD interaction energies for the benzene sandwich dimer. The bold dashed curves show the MEPs along each potential. (bottom) energies along the corresponding MEPs. SAPT2+3(δMP2)/aug-cc-pVTZ is generally considered the 'gold standard' among SAPT methods.[1] We have used the more economical SAPT2+3/def2-TZVPD, which introduces negligible error in the case of the benzene dimer: the RMSE along the MEP is 0.02 kcal/mol.

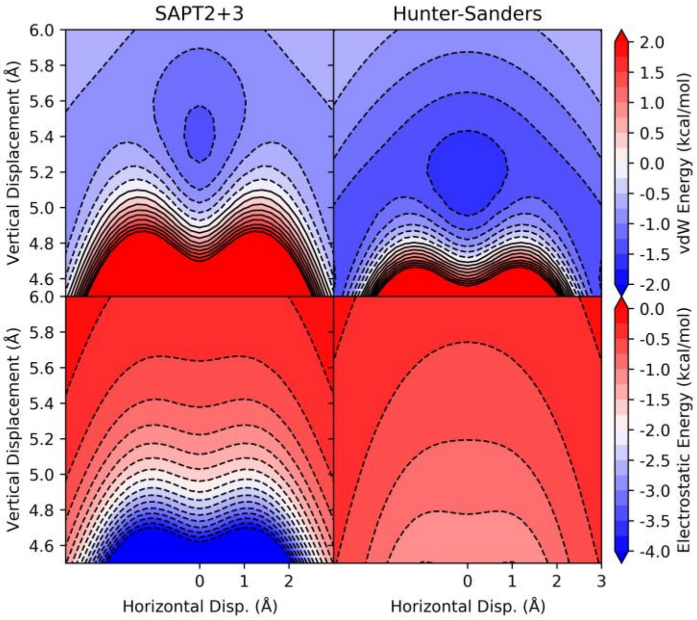

Figure S2. vdW (top) and electrostatic (bottom) contribution to the interaction energy of the T-shaped benzene dimer from SAPT2+3 (left) and the Hunter-Sanders potential (right).

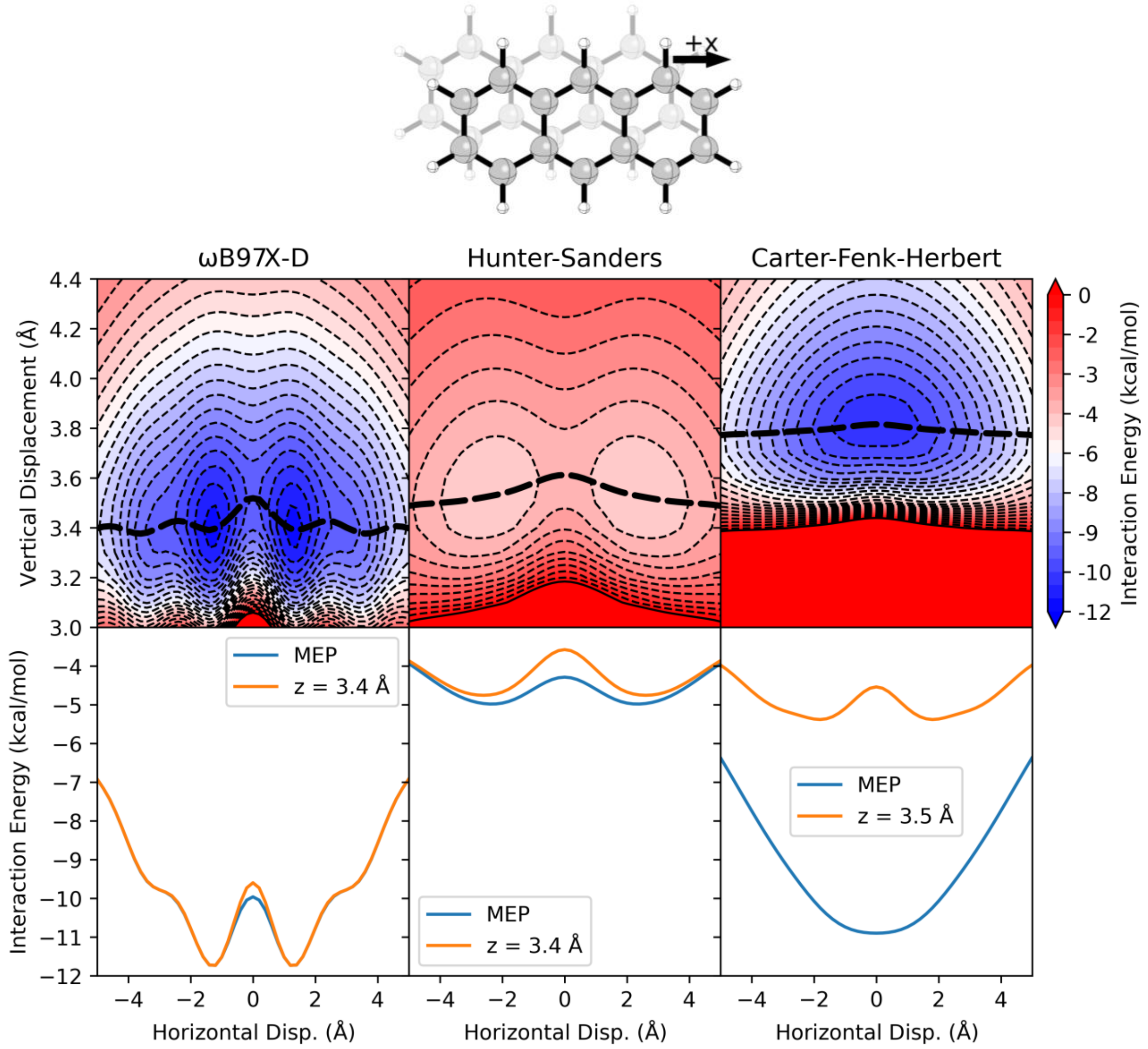

Figure S3. Interaction energies from (left) wB97X-D/def2-TZVP, (middle) the Hunter-Sanders potential (Eq. 1), and (right) the Carter-Fenk-Herbert potential (Eq. 2) for the parallel-stacked anthracene dimer displaced 1.0 A along the y-axis (pictured above). The bold dashed curve shows the MEP on the corresponding surface. The energies along the MEP and at constant z-values are shown in the bottom panels.

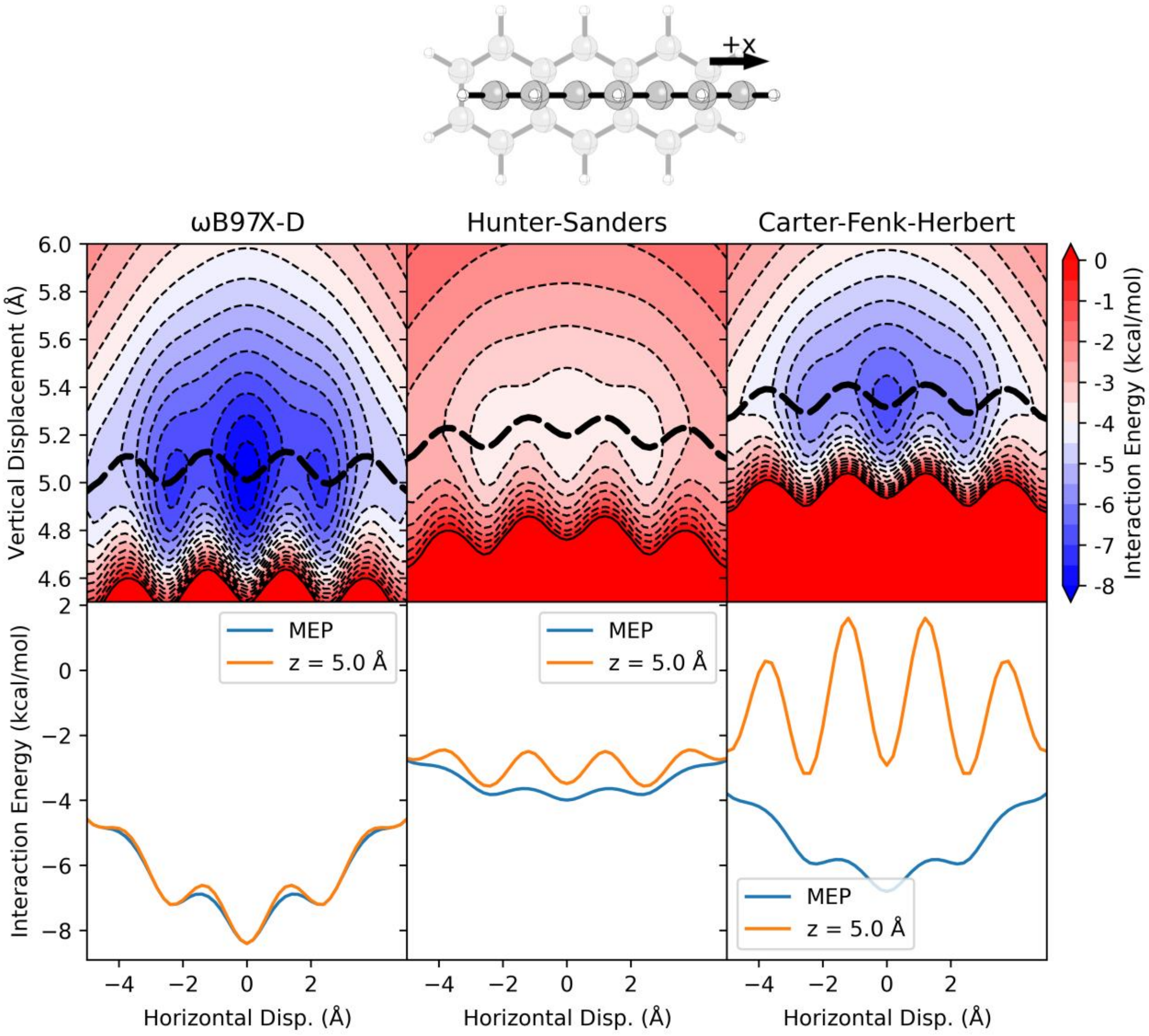

Figure S4. Interaction energies from (left) wB97X-D/def2-TZVP, (middle) the Hunter-Sanders potential (Eq. 1), and (right) the Carter-Fenk-Herbert potential (Eq. 2) for the T-shaped anthracene dimer (pictured above). The bold dashed curve shows the MEP on the corresponding surface. The energies along the MEP and at constant z-values are shown in the bottom panels.

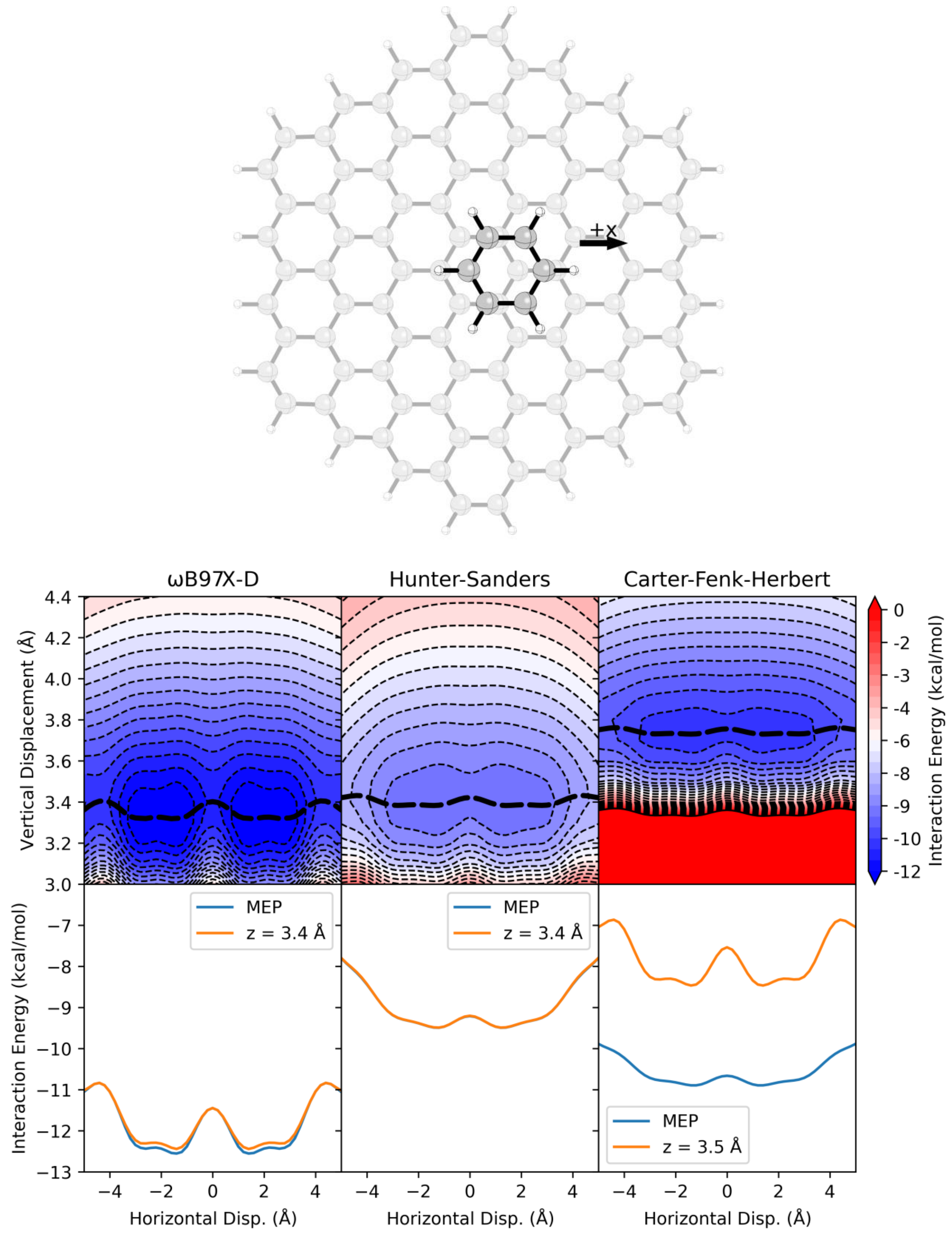

Figure S5. Interaction energies from (left) wB97X-D/def2-TZVP, (middle) the Hunter-Sanders potential (Eq. 1), and (right) the Carter-Fenk-Herbert potential (Eq. 2) for the parallel-stacked

benzene-$C_{96}H_{24}$ dimer (pictured above). The bold dashed curve shows the MEP on the corresponding surface. The energies along the MEP and at constant z-values are shown in the bottom panels.

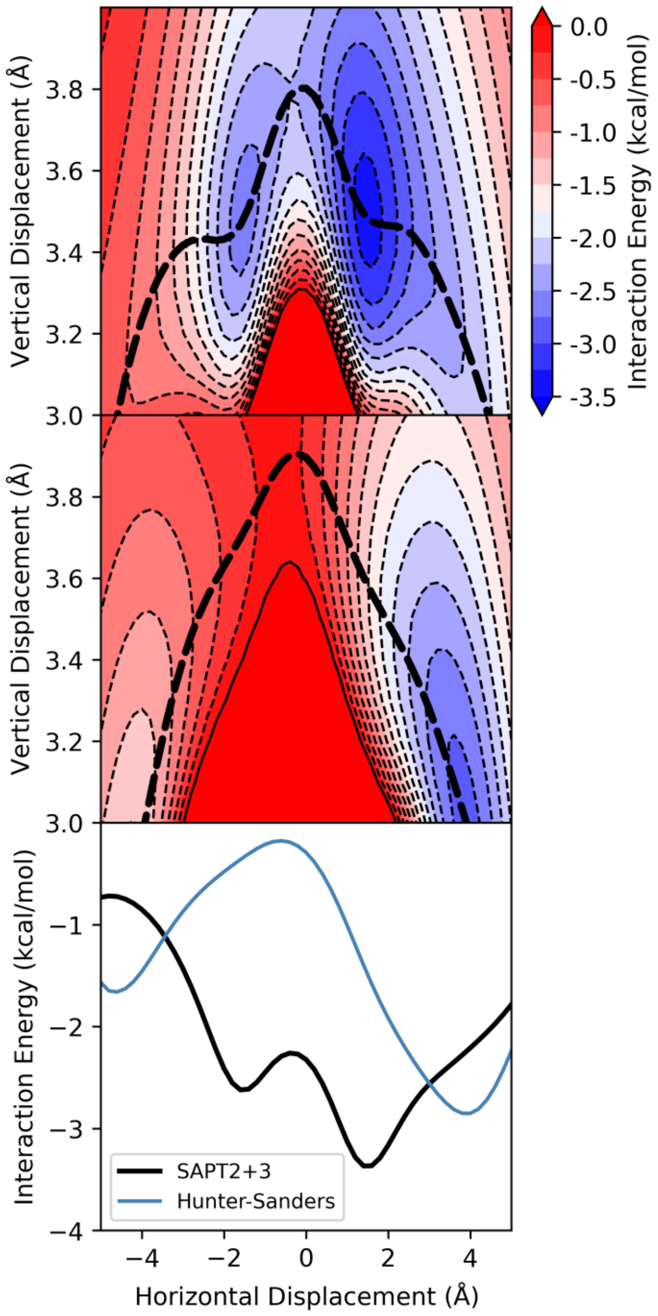

Figure S6. Interaction energies from (top) SAPT and (middle) the Hunter-Sanders potential (Eq 1) for the parallel-stacked benzene-pyridine dimer. The bold dashed curve shows the MEP on the corresponding surface. (bottom) Interactions along the corresponding MEPs.

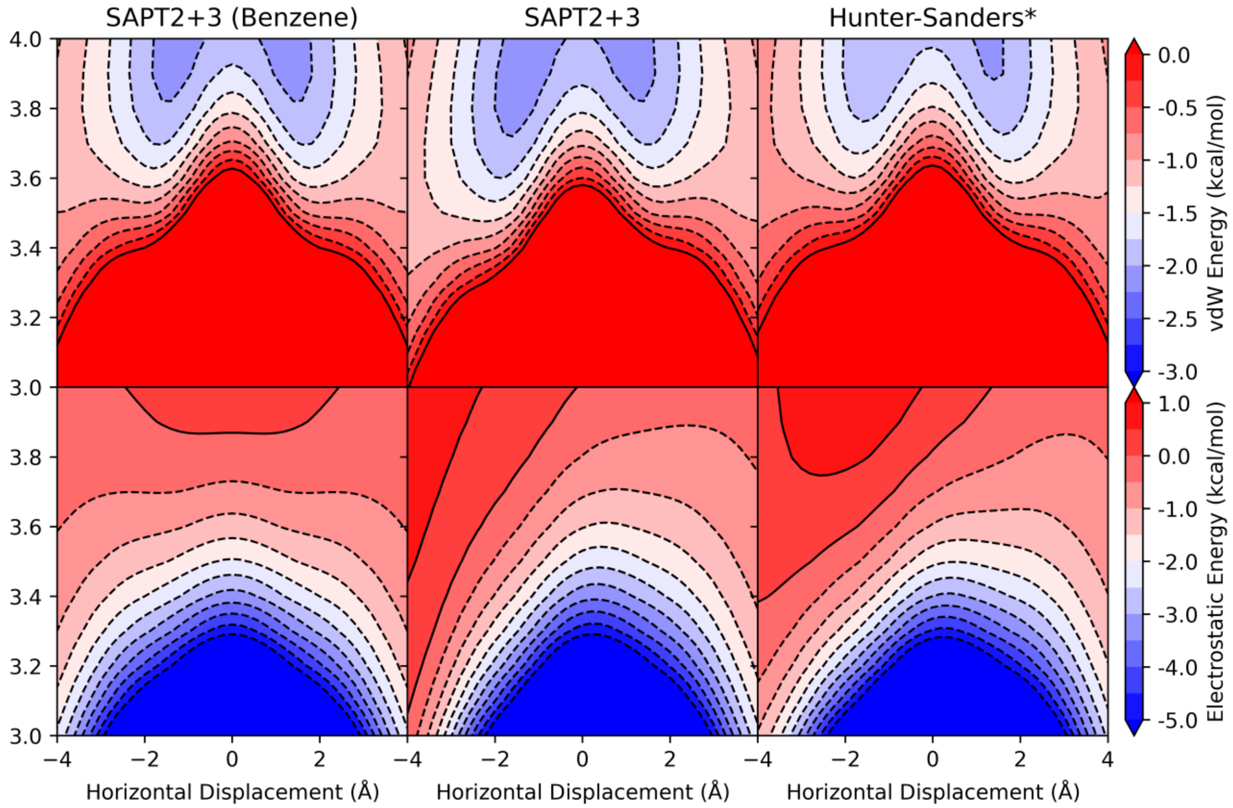

Figure S7. vdW (top) and electrostatic (bottom) contribution to the interaction energy of the (left) parallel benzene-pyridine dimer computed with SAPT2+3 and the benzene-benzonitrile dimer from (middle) SAPT2+3 and (right) Eq. 3 (Hunter-Sanders*).

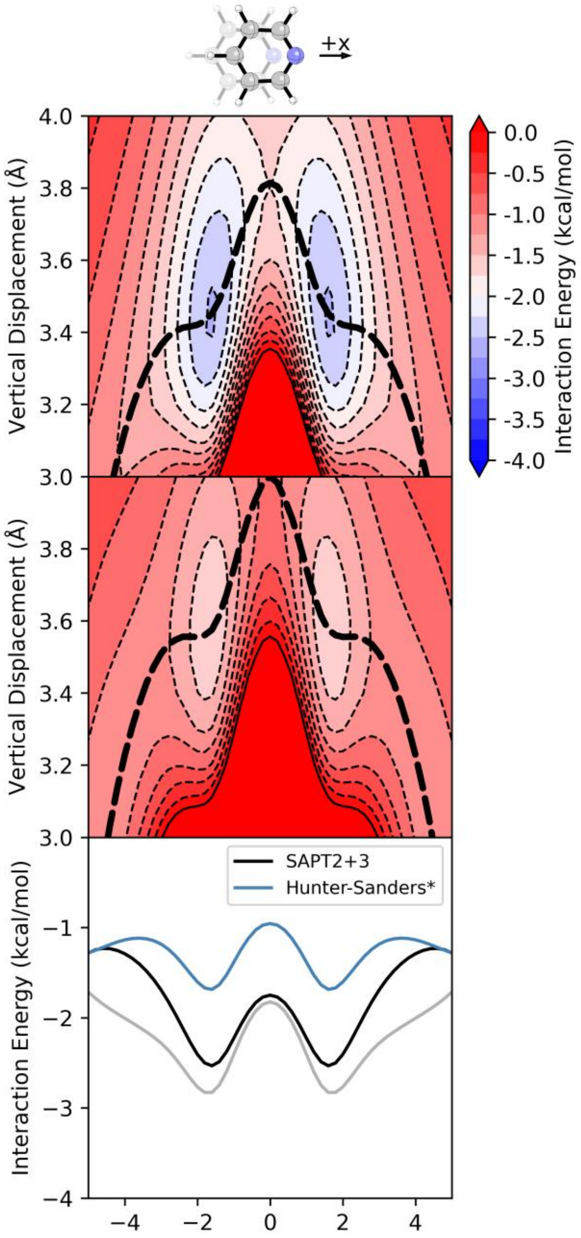

Figure S8. Interaction energies from (top) SAPT and (bottom) the Hunter-Sanders potential for the aligned pyridine dimer. The bold dashed curve shows the MEP on the corresponding surface. The light gray curve is the SAPT2+3 MEP for the benzene dimer, for reference.

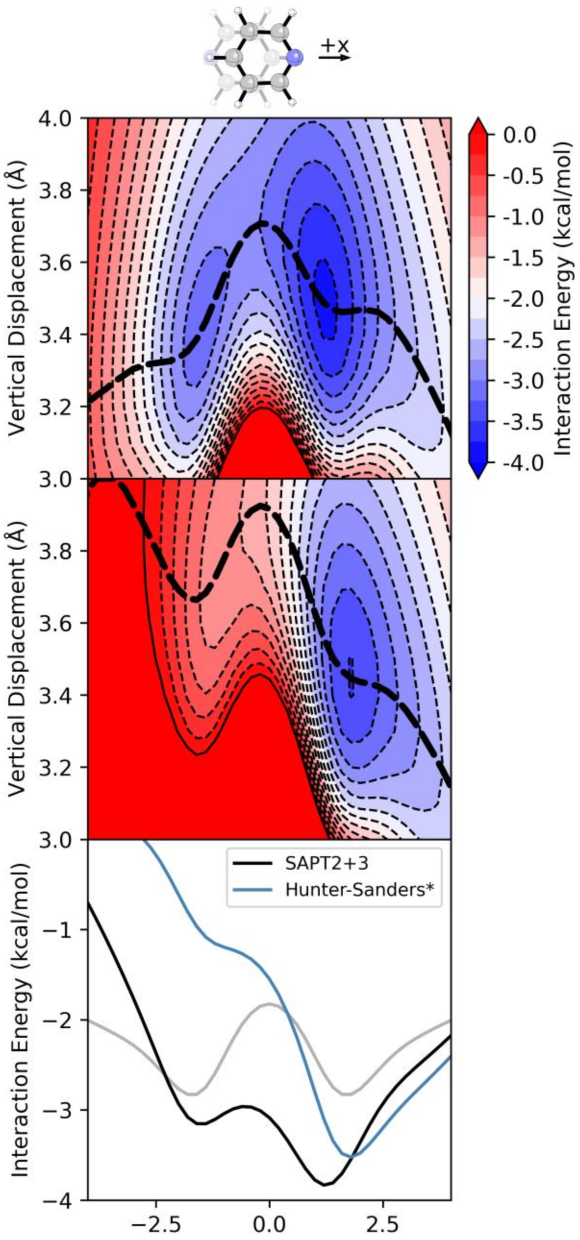

Figure S9. Interaction energies from (top) SAPT and (bottom) the Hunter-Sanders potential for the anti-aligned pyridine dimer. The bold dashed curve shows the MEP on the corresponding surface. The light gray curve is the SAPT2+3 MEP for the benzene dimer, for reference.

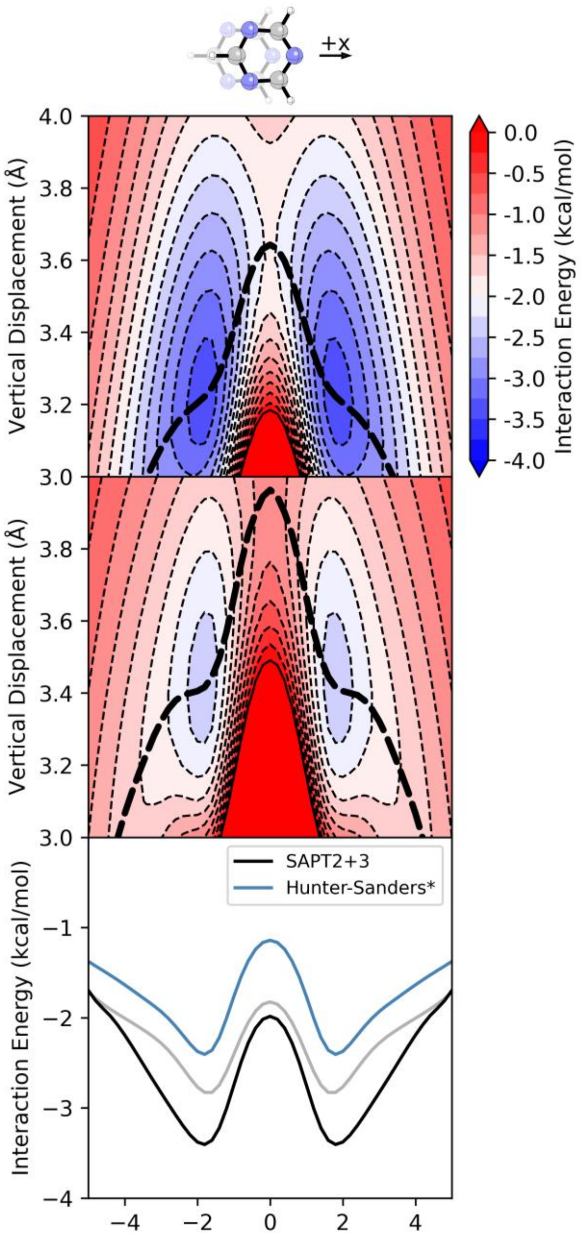

Figure S10. Interaction energies from (top) SAPT and (bottom) the Hunter-Sanders potential for the aligned s-triazine dimer. The bold dashed curve shows the MEP on the corresponding surface. The light gray curve is the SAPT2+3 MEP for the benzene dimer, for reference.

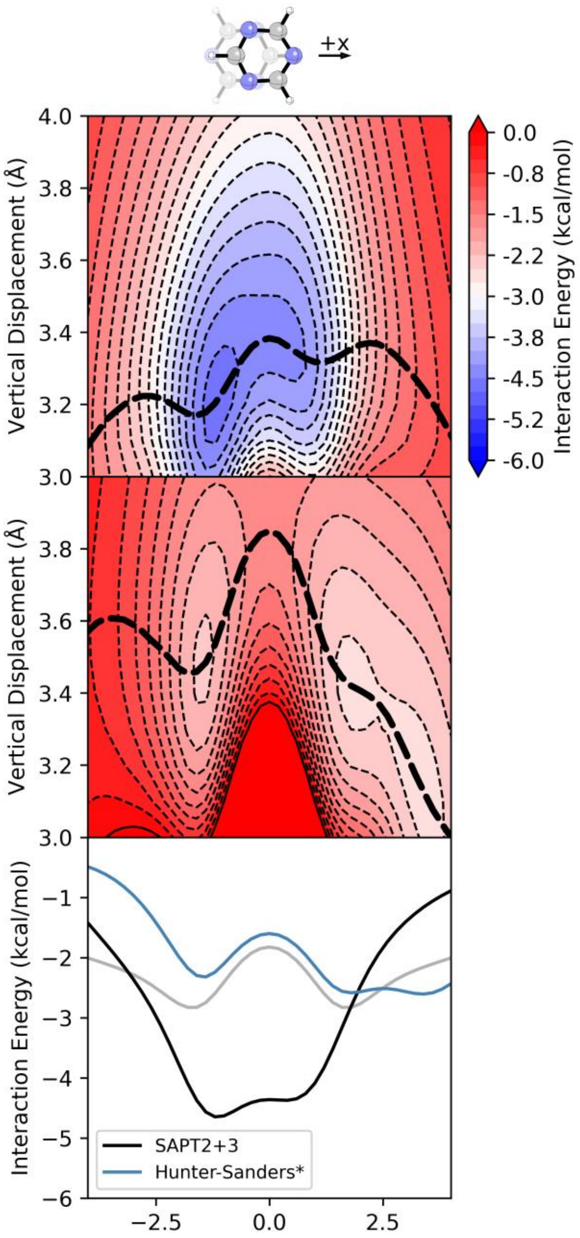

Figure S11. Interaction energies from (top) SAPT and (bottom) the Hunter-Sanders potential for the anti-aligned s-triazine dimer. The bold dashed curve shows the MEP on the corresponding surface. The light gray curve is the SAPT2+3 MEP for the benzene dimer, for reference.

Table S1. Interaction energies and interaction energies relative to X = H for $C_6H_5X\cdots C_6H_6$ sandwich dimers computed using Eq. 1 along with SAPT2+3 data, in kcal/mol.

	Hunter-Sanders (Eq. 1)		SAPT2+3	
X	E_{int}	E_{int}^{rel}	E_{int}	E_{int}^{rel}
CCH	-0.8	-0.6	-2.7	-1.0
CH_2OH	-0.8	-0.6	-2.4	-0.6
CH_3	-0.5	-0.4	-2.3	-0.6
CHO	-1.1	-0.9	-2.9	-1.2
CN	-1.3	-1.2	-3.3	-1.5
$COCH_3$	-1.2	-1.0	-3.1	-1.3
$COOCH_3$	-1.4	-1.2	-3.0	-1.2
COOH	-1.4	-1.3	-3.0	-1.2
**H**	**-0.2**	**0.0**	**-1.8**	**0.0**
$N(CH_3)_2$	-0.8	-0.6	-2.6	-0.8
NH_2	-0.4	-0.3	-2.1	-0.3
$NHCH_3$	-0.6	-0.4	-2.4	-0.6
NHOH	-0.8	-0.6	-2.5	-0.8
NO	-1.2	-1.0	-3.1	-1.3
NO_2	-2.1	-2.0	-3.6	-1.9
OH	-0.5	-0.4	-2.2	-0.4
OMe	-0.6	-0.4	-2.4	-0.6

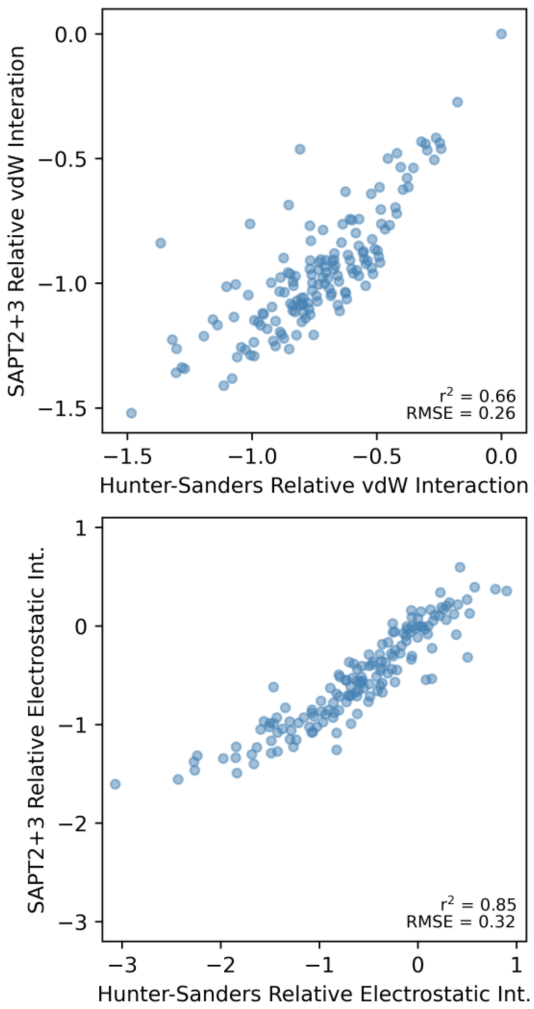

Figure S12. Correlation of vdW (top) and electrostatic (bottom) components (in kcal/mol) to the interaction energy of $C_6H_5X\cdots C_6H_5Y$ sandwich dimers from SAPT2+3 and the Hunter-Sanders potential.

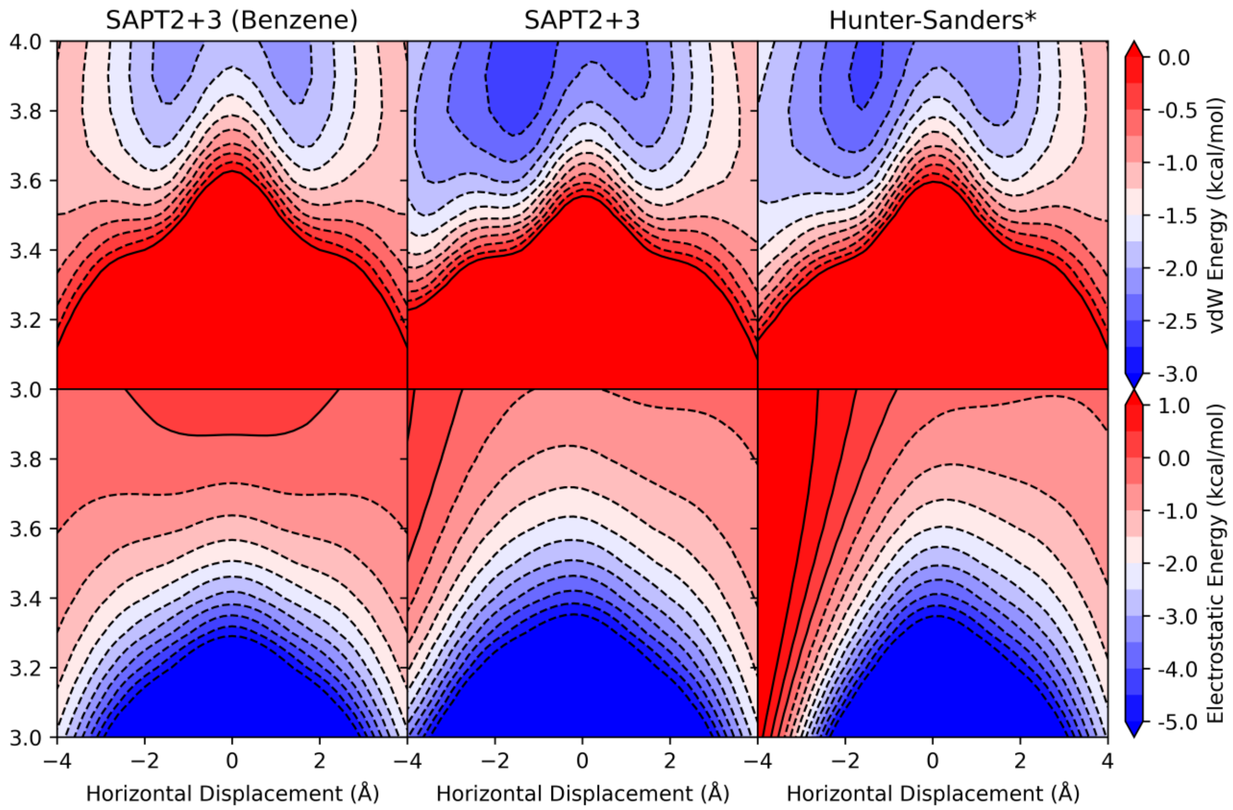

Figure S13. vdW (top) and electrostatic (bottom) contribution to the interaction energy of the (left) parallel benzene-benzene dimer computed with SAPT2+3 and the benzene-benzonitrile dimer from (middle) SAPT2+3 and (right) Eq. 3 (Hunter-Sanders*).

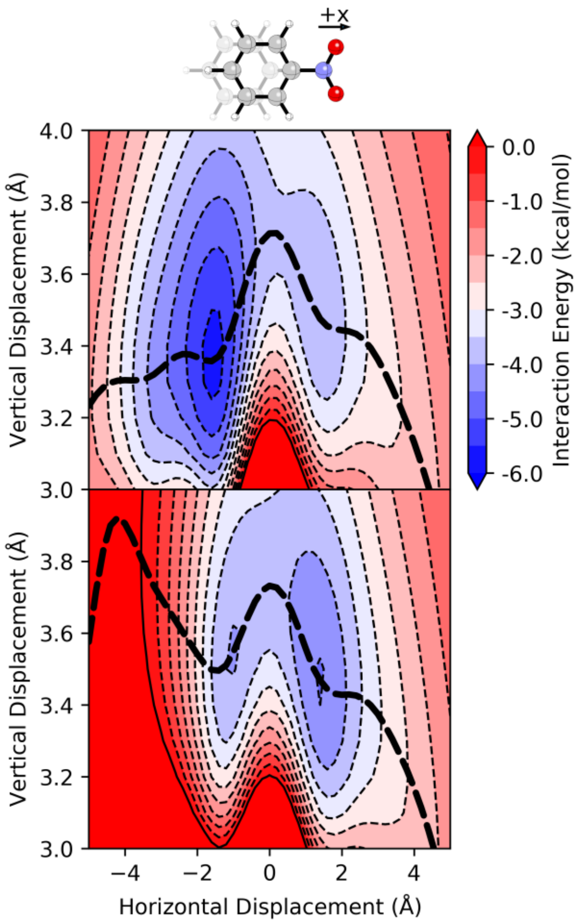

Figure S14. Interaction energies from (top) SAPT and (bottom) the Hunter-Sanders potential for the benzene-nitrobenzene dimer. The bold dashed curves shows the MEP on the corresponding surface.

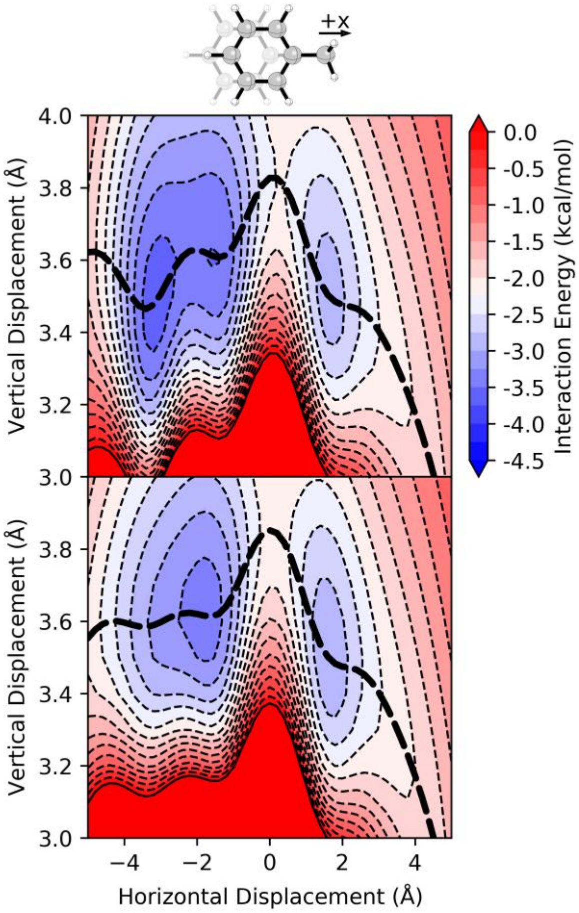

Figure S15. Interaction energies from (top) SAPT and (bottom) the Hunter-Sanders potential for the benzene-toluene dimer. The bold dashed curves shows the MEP on the corresponding surface.

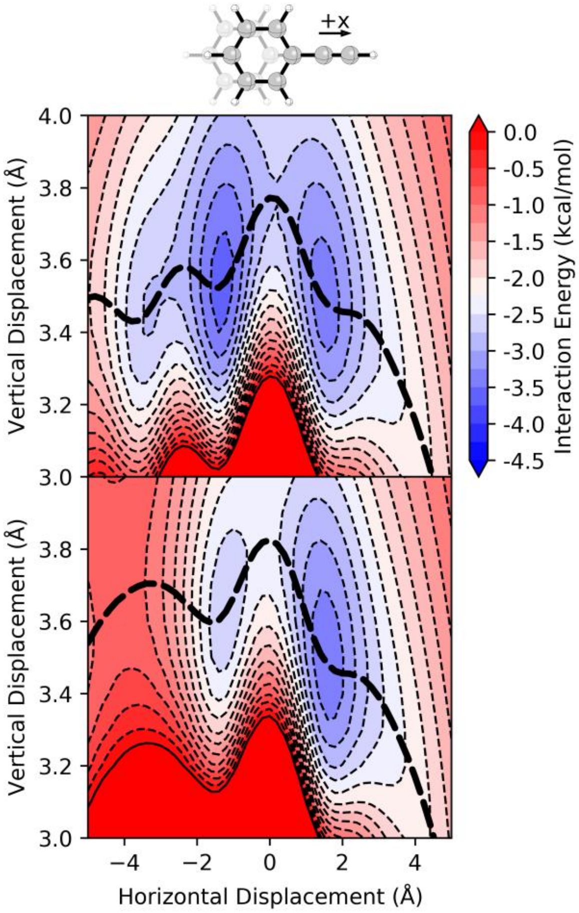

Figure S16. Interaction energies from (top) SAPT and (bottom) the Hunter-Sanders potential for the benzene-benzonitrile dimer. The bold dashed curves shows the MEP on the corresponding surface.

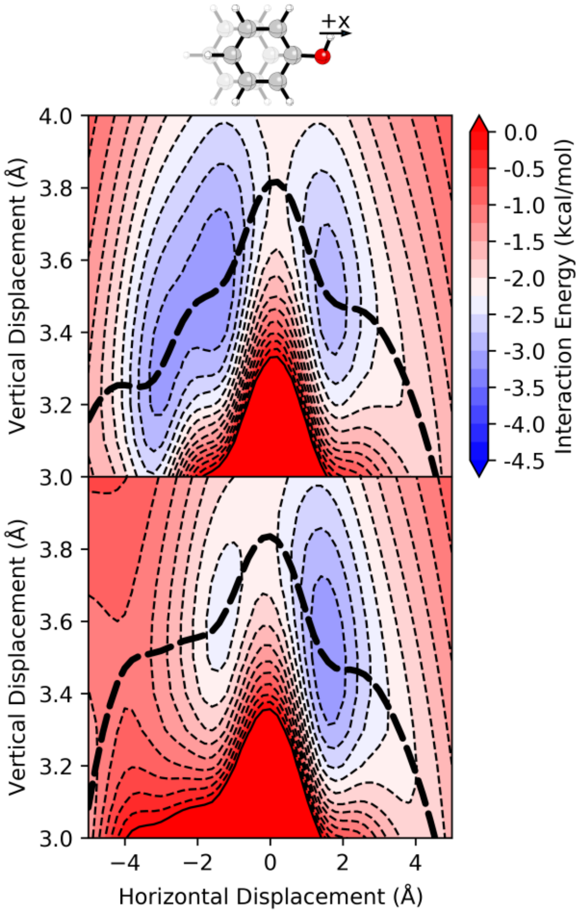

Figure S17. Interaction energies from (top) SAPT and (bottom) the Hunter-Sanders potential for the benzene-phenol dimer. The bold dashed curves shows the MEP on the corresponding surface.

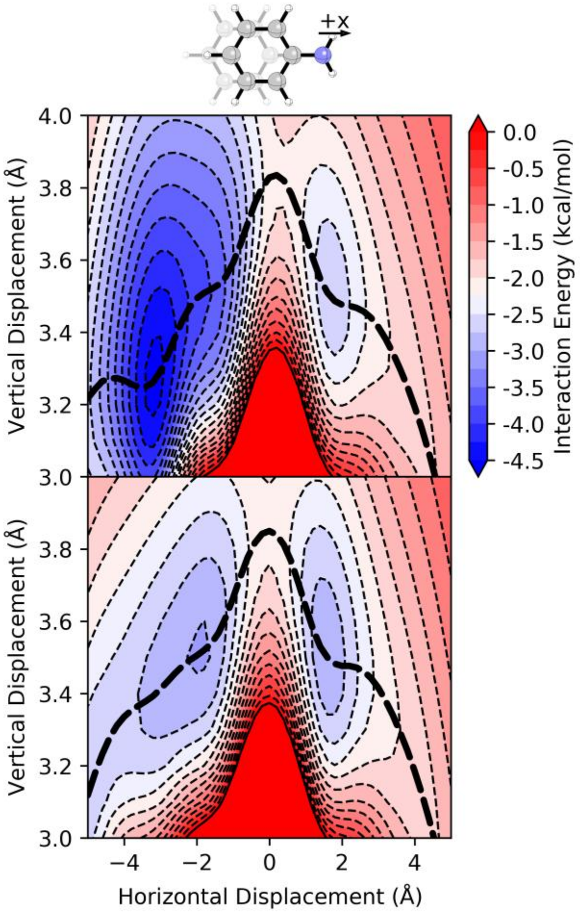

Figure S18. Interaction energies from (top) SAPT and (bottom) the Hunter-Sanders potential for the benzene-aniline dimer. The bold dashed curves shows the MEP on the corresponding surface.

S2. Implementation of Eq. 1 (Hunter-Sanders) and Eq. 2 (Carter-Fenk-Herbert)

Eqs. 1 and 2 were implemented for general planar aromatic systems using AaronTools.[2] Details are provided here for reference. The Hunter-Sanders model used the electrostatic model described in the main text paired with the following vdW potential from Caillet and Claverie,[3] where the energy is in kcal/mol and R_{ij} is the distance (in Angstroms) between atoms *i* and *j*:

$$E_{vdW} = \sum_{i \in A} \sum_{j \in B} k_i k_j \left(C e^{-\alpha z_{ij}} - \frac{A}{z_{ij}^6} \right)$$

$$z_{ij} = \frac{R_{ij}}{2\sqrt{R_i R_j}}$$

$$A = 0.14$$

$$C = 47000$$

$$\alpha = 12.35$$

k_i and k_j are element-specific scaling constants and R_i and R_j are van der Waals radii "generally taken from Bondi":[4]

	k_i	R_i (in Å)
H	1	1.2
C	1	1.77 (aromatic)
		1.70 (aliphatic)
N	1.18	1.6 (aromatic)
O	1.36	1.5 (aromatic)

Radii are provided for both aromatic and aliphatic C atoms but only aromatic N and O atoms. We explored using standard Bondi radii (1.70, 1.55, and 1.52 Å for C, N, and O) for the substituents in the substituted benzene dimers, but it made little difference. It is also unclear whether the C and N in a nitrile group, for example, are better represented as aliphatic or aromatic atoms. For simplicity, the data in the main text is based on the use of the aromatic R_i-values for all elements.

As recently noted by Herbert *et al.*,[5] Ref. [6] does not provide the values of the σ- and π-charges that were used. It seems that where available, Hunter and Sanders used values taken from previous work, but the only specific references they provide are for porphyrins. In these cases, the charges were based on Mulliken population analyses of either iterative extended Huckel (IEH) or CNDO/2 wavefunctions. In 1991, Hunter *et al.*[7] noted that the original 1990 paper[6] used (σ, π) charges on C of (0.95, 0.5) and a charge on H of 0.05 for benzene, but do not explain how these were computed. After exploring several simple QM wavefunctions, the best agreement we could obtain was by performing Mulliken population analysis on a HF/STO-3G

wavefunction, which gives (σ, π) charges on C of (0.94, 0.5) and a charge on H of 0.06. As such, in the main text we use the charges from Ref [7] for benzene, except for Figure 9. For benzene in Figure 9, as well as all other systems, we obtain charges from Mulliken population analysis of a HF/STO-3G wavefunction computed using Psi4[8] as follows. In Mulliken population analysis, the total atomic charge for atom A is

$$q^A = Z^A - \sum_{\mu \in A} (\mathbf{DS})_{\mu\mu}$$

where Z^A is the nuclear charge on atom A, the sum runs over basis functions on atom A, and **D** and **S** are the density and overlap matrices, respectively. For atoms that are part of the π-system (*e.g.* *sp* and sp^2-hybridized atoms), this total charge is partitioned into π- and σ-charges as:

$$q_\pi^A = -\frac{1}{2} \sum_{\mu \in A}^{p_z} (\mathbf{DS})_{\mu\mu}$$

$$q_\sigma^A = q^A + 2q_\pi^A$$

where the sum in the q_π^A definition is restricted to p_z-type (assuming the molecule is in the *xy*-plane) basis functions. For hydrogen atoms, as well as atoms not contributing to the π-system (*e.g.* the methyl C in toluene or anisole), $q_\sigma^A = q^A$ and $q_\pi^A = 0$. We note that the amino substituents in aniline, *N*-methylaniline, *etc.* are slightly pyramidalized; however, we treated these as an sp^2-hybridized atoms for application of the Hunter-Sanders model.

For the empirical potential from Carter-Fenk and Herbert,[9] E_{pauli}^{CFH} was implemented exactly as described in the SI of Ref. [9] (using the original Bondi radii).[4] E_{disp}^{aiD3} was implemented as described in the SI of Ref. [10]. Results from this potential agree to 0.001 kcal/mol with data provided by Carter-Fenk and Herbert.

S3. Origin of the Scaling Parameter in the Carter-Fenk-Herbert vdW Potential

E_{pauli}^{CFH} contains an empirical constant (η = 0.36) that scales the vdW radii. Although Carter-Fenk and Herbert do not disclose how this value was chosen, they do indicate in Ref [11] that this model was parameterized for the benzene dimer. Figure S19 shows the vdW component of XSAPT-MBD/def2-TZVPD interaction energies from Herbert *et al.*[12] for the benzene dimer as a function of lateral displacements for z = 3.1, 3.5 and 3.9 Å. Also shown are interaction energies from E_{int}^{CFH} using η = 0.35 - 0.38 at each of these vertical separations. The optimal value of η is dependent on the vertical separation of the rings, with η = 0.35 optimal for *z* = 3.1 Å, η = 0.36

for z = 3.5 Å, and somewhere between η = 0.37 and 0.38 for z = 3.9 Å. Carter-Fenk and Herbert chose η = 0.36 and then plotted data for parallel-stacked benzene-benzene and benzene-hexafluorobenzene at z = 3.5 Å.[9]

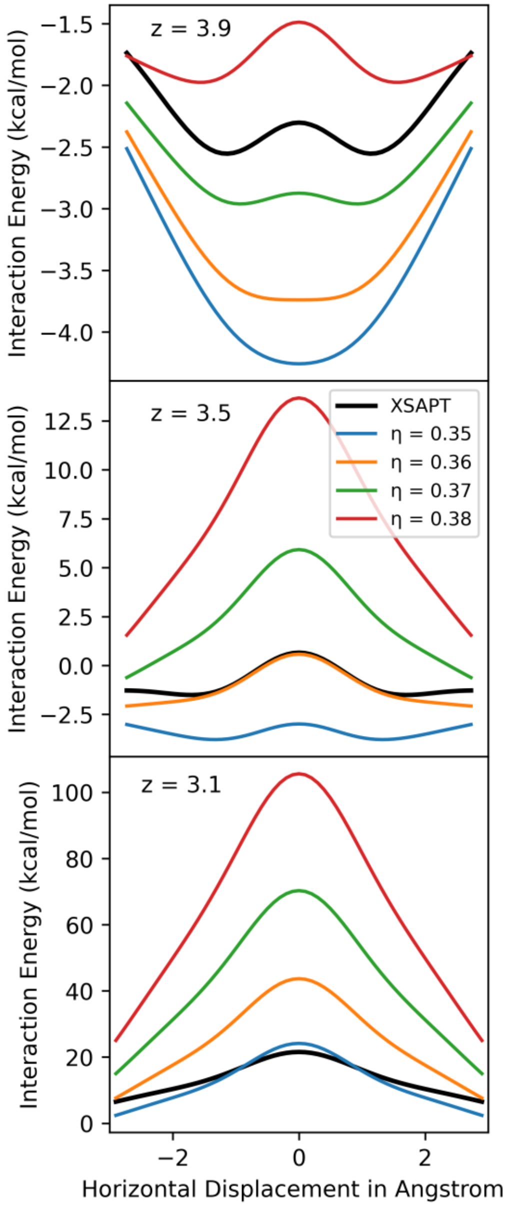

Figure S19. Interaction energies from XSAPT-MBD[12] and the Carter-Fenk-Herbert potential (Eq 1) using different values of the scaling parameter η for the parallel-stacked benzene dimer at z = 3.9 (top), 3.5 (middle) and 3.1 Å (bottom).

S4. Carter-Fenk-Herbert Potential Applied to Heterocyclic and Substituted Systems

Although Herbert *et al*.[11] have made the broad statement that "These results inspired us to develop an alternative semiclassical model (to replace eq [1]) in which we dispense with electrostatics altogether, replace the dispersion model with aiD3, and replace the exponential repulsion model with one based on the overlap of atom-centered spherical Gaussian functions," it seems that this was only meant to apply in the context of aromatic hydrocarbons. Indeed, more recent work from Herbert *et al*.[12] has shown that even accurately computed vdW potentials are unable to capture many of the salient features of the interaction potentials for stacked heterocyclic dimers and those of substituted benzenes. As such, one would not expect the Carter-Fenk-Herbert potential (Eq 2), which provides a simple approximate vdW potential and neglects electrostatic effects, to provide an accurate description of such systems. As such, in the main text we only evaluated Eq. 2 for the stacked aromatic hydrocarbons for which it seems to have been designed. However, for completeness (and at the request of a reviewer), we show limited results here of the application of the Carter-Fenk-Herbert potential to the benzene-pyridine dimer as well as monosubstituted benzene dimers.

For the parallel-stacked benzene-pyridine dimer (Figure S20), Eq. 2 predicts an interaction energy surface with single minimum, although with a small degree of asymmetry. That is, as seen for the benzene dimer, the Carter-Fenk-Herbert potential is unable to capture the double-well shape characteristic of this dimer; moreover, it drastically underestimates the impact of the N-heteroatom on the potential. Again, this is expected, because the impact of N-atoms on stacking interactions is mostly electrostatic in origin.[12]

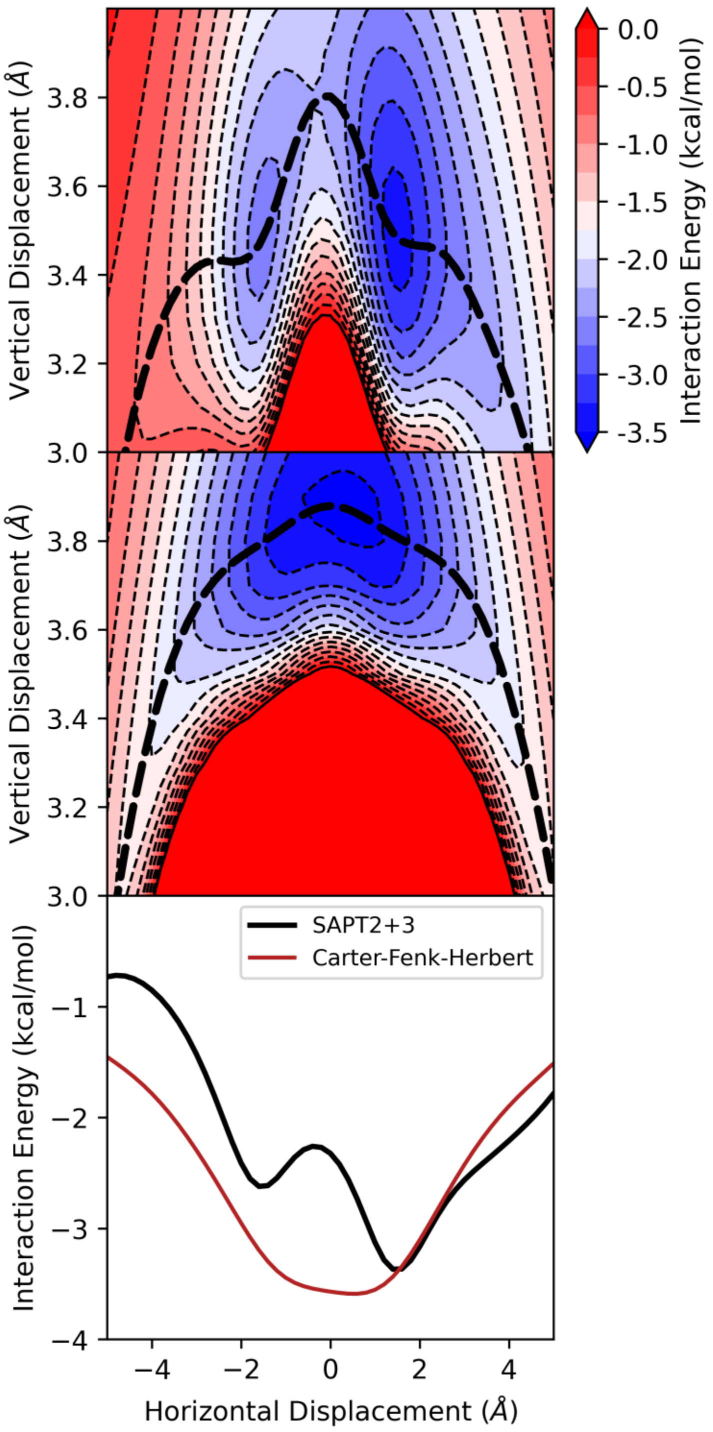

Figure S6. Interaction energies from (top) SAPT and (middle) the Carter-Fenk-Herbert potential (Eq 2) for the parallel-stacked benzene-pyridine dimer. The bold dashed curve shows the MEP on the corresponding surface. (bottom) Interactions along the corresponding MEPs.

If one instead applies an analogue of Eq. 3, in which the difference in interaction energy between the benzene-pyridine and benzene-benzene dimer, predicted using the Carter-Fenk-Herbert potential, is added to an accurate potential for the benzene dimer, the performance is even worse

(see Figure S21). In particular, Eq. 2 is unable to capture the stabilizing impact of the N-atom for +*x* displacements (in which this atom is moved away from the other ring) and predicts an unphysical enhancement for negative displacements. This appears to stem from the unphysical behavior of Eq. 2 for intermonomer separations below 3.5 Å, which leads to drastic differences between the benzene-benzene and benzene-pyridine interaction energies for -*x* displacments.

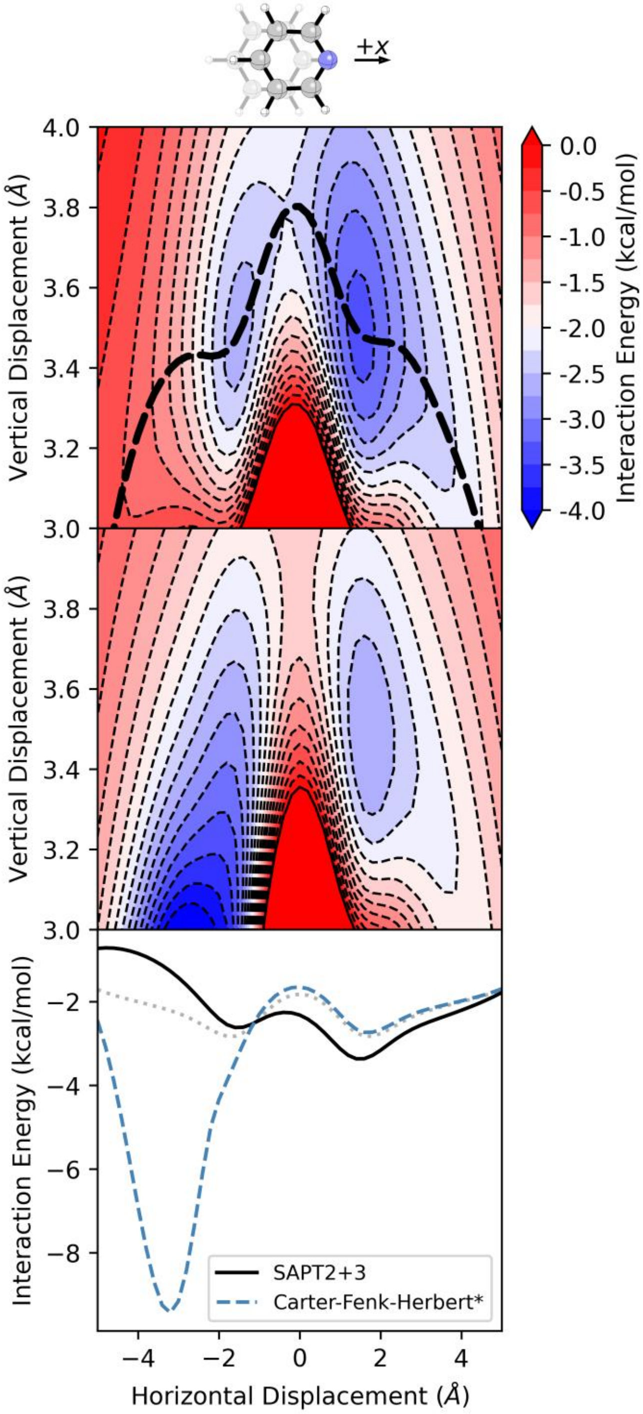

Figure 7. Interaction energy for the parallel-stacked pyridine-benzene dimer as a function of lateral and vertical displacements from (top) SAPT2+3 and (middle) analogue of Eq. 3 using the Carter-Fenk-Herber potential. The dashed line is the MEP across the corresponding potential. (bottom) Interaction energy along the corresponding MEP computed using SAPT2+3 and Carter-Fenk-Herbert*. The dotted light gray curve is the SAPT2+3 MEP for the benzene dimer, for reference.

Finally, in terms of monosubstituted benzene sandwich dimers, Eq. 2 provides no predictive capability in terms of interaction energies relative to the benzene dimer (r^2 = 0.01; see Figure S22). Again, this is not surprising, given the well-established importance of electrostatic effects in substituent effects in stacking interactions.

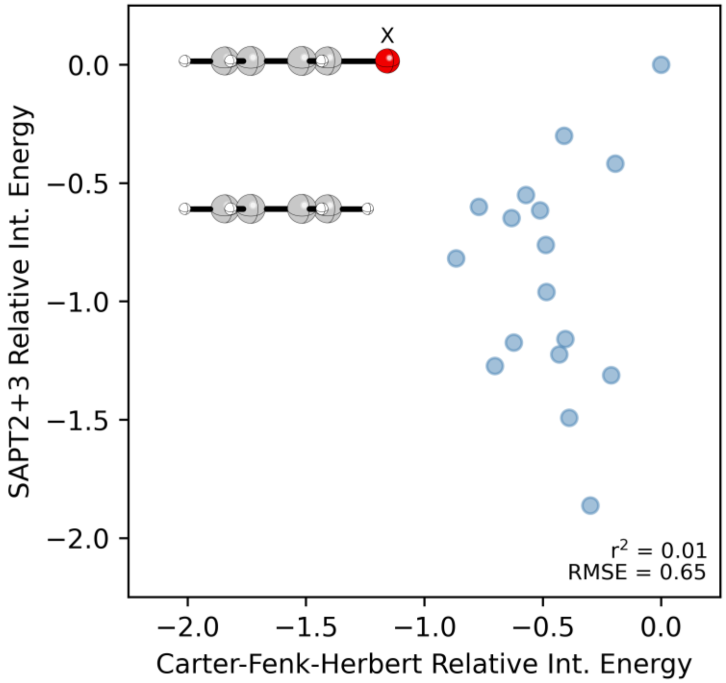

Figure S22. Correlation of SAPT2+3/def2-TZVP interaction energies (in kcal/mol) with those predicted from the Carter-Fenk-Herbert potential (Eq. 2) for monosubstituted benzene dimers.

S5. Computational Details

Two-dimensional potential energy surfaces were computed by scanning over horizontal and vertical distances in increments of 0.2 and 0.1 Å, respectively, with monomer geometries frozen. Minimum energy paths (MEPs) were evaluated by finding the energy minimum along a one-dimensional cubic spline interpolator fit to the interaction energies at each horizontal displacement. For the substituted systems in Figure 9, monomer geometries were taken from Ref. [13] For the $C_6H_5X\cdots C_6H_6$ sandwich dimers, optimal vertical separations were taken from Ref. [13]. For the $C_6H_5X\cdots C_6H_5Y$ sandwich dimers all vertical separations were 4 Å, as in Ref. [13]. For all other systems (including the monosubstituted dimers in 10 and 11, monomer geometries were optimized at the wB97X-D/def2-TZVP level of theory.[14, 15] For anthracene dimers as well as the dimer of benzene with $C_{96}H_{24}$, interaction energies from the Hunter-Sanders and Carter-Fenk-Herbert potentials were compared with data computed at the wB97X-D/def2-TZVP level of theory.[14, 15] All DFT computations were performed using Gaussian 16.[16]

S6. Other Data

Geometries for all monomers are available in `SI_aromatics.xyz` and `SI_Figure9.xyz`. The latter contains the geometries from in Ref. [13] for the monosubstituted benzenes in Figure 9. The former contains optimized geometries for all other systems, including the the monosubstituted systems in Figures 10 and 11. SAPT energy components and energies from Eq. 1 and Eq. 2 (where applicable) are available in `SI_Hunter_Sanders.xls`. Finally, the σ- and π-charges for all systems can be found in `SI_aromatics_charges.txt` and `SI_Figure9_charges.txt` along with the atomic coordinates (columns 1-4) for reference. Column 5 is q_σ and column 6 is q_π.

References

(1) Parker, T. M.; Burns, L. A.; Parrish, R. M.; Ryno, A. G.; Sherrill, C. D. Levels of symmetry adapted perturbation theory (SAPT). I. Efficiency and performance for interaction energies. *J. Chem. Phys.* **2014**, *140*, 094106.

(2) Ingman, V. M.; Schaefer, A. J.; Andreola, L. R.; Wheeler, S. E. QChASM: Quantum chemistry automation and structure manipulation. *WIREs Computational Molecular Science* **2020**, *11* (4), e1510.

(3) Caillet, J.; Claverie, P. Theoretical evaluations of the intermolecular interaction energy of a crystal: application to the analysis of crystal geometry. *Acta Crystallographica Section A* **1975**, *31* (4), 448-461.

(4) Bondi, A. van der Waals Volumes and Radii. *The Journal of Physical Chemistry* **2002**, *68* (3), 441-451.

(5) Carter-Fenk, K.; Lao, K. U.; Herbert, J. M. Correction to "Predicting and Understanding Noncovalent Interactions Using Novel Forms of Symmetry-Adapted Perturbation Theory". *Acc Chem Res* **2025**, *58* (6), 1051-1053.

(6) Hunter, C. A.; Sanders, J. K. M. The Nature of π-π Interactions. *J. Am. Chem. Soc.* **1990**, *112*, 5525-5534.

(7) Hunter, C. A.; Singh, J.; Thornton, J. M. Pi-pi interactions: the geometry and energetics of phenylalanine-phenylalanine interactions in proteins. *J Mol Biol* **1991**, *218* (4), 837-846.

(8) Turney, J. M.; Simmonett, A. C.; Parrish, R. M.; Hohenstein, E. G.; Evangelista, F. A.; Fermann, J. T.; Mintz, B. J.; Burns, L. A.; Wilke, J. J.; Abrams, M. L.; et al. PSI4: an open-source ab initio electronic structure program. *Wires Comput Mol Sci* **2012**, *2* (4), 556-565.

(9) Carter-Fenk, K.; Herbert, J. M. Electrostatics does not dictate the slip-stacked arrangement of aromatic pi-pi interactions. *Chem Sci* **2020**, *11* (26), 6758-6765.

(10) Lao, K. U.; Herbert, J. M. Accurate and efficient quantum chemistry calculations for noncovalent interactions in many-body systems: the XSAPT family of methods. *J Phys Chem A* **2015**, *119* (2), 235-252.

(11) Carter-Fenk, K.; Lao, K. U.; Herbert, J. M. Predicting and Understanding Non-Covalent Interactions Using Novel Forms of Symmetry-Adapted Perturbation Theory. *Acc Chem Res* **2021**, *54* (19), 3679-3690.

(12) Schramm, B.; Gray, M.; Herbert, J. M. Substituent and Heteroatom Effects on pi-pi Interactions: Evidence That Parallel-Displaced pi-Stacking is Not Driven by Quadrupolar Electrostatics. *J Am Chem Soc* **2025**, *147* (4), 3243-3260.
(13) Wheeler, S. E. Local Nature of Substituent Effects in Stacking Interactions. *J. Am. Chem. Soc.* **2011**, *133*, 10262-10274.
(14) Chai, J. D.; Head-Gordon, M. Long-range corrected hybrid density functionals with damped atom-atom dispersion corrections. *Phys Chem Chem Phys* **2008**, *10* (44), 6615-6620, 10.1039/B810189B.
(15) Weigend, F.; Ahlrichs, R. Balanced basis sets of split valence, triple zeta valence and quadruple zeta valence quality for H to Rn: Design and assessment of accuracy. *Phys. Chem. Chem. Phys.* **2005**, *7*, 3297-3305.
(16) *Gaussian 16 Rev. C.01*; Wallingford, CT, 2016.
